# An experimental medicine study of the effects of simvastatin on emotional processing, reward learning, verbal memory, and inflammation in healthy volunteers

**DOI:** 10.1007/s00213-022-06156-y

**Published:** 2022-05-05

**Authors:** Riccardo De Giorgi, Alice M. G. Quinton, Shona Waters, Philip J. Cowen, Catherine J. Harmer

**Affiliations:** 1 Department of Psychiatry, University of Oxford, Warneford Hospital, Warneford Lane, Oxford, OX3 7JX Oxfordshire UK; 2 Oxford Health NHS Foundation Trust, Warneford Hospital, Warneford Lane, Oxford, OX3 7JX Oxfordshire UK

**Keywords:** Simvastatin, Emotional processing, Reward learning, Verbal memory, Inflammation, Experimental medicine, Precision psychiatry, Healthy volunteers, Depression

## Abstract

**Rationale:**

Clinical studies suggest that the highly lipophilic, anti-inflammatory molecule, simvastatin, might be an ideal candidate for drug repurposing in the treatment of depression. The neuropsychological effects of simvastatin are not known, but their ascertainment would have significant translational value about simvastatin’s influence on mood and cognition.

**Objectives:**

We aimed to investigate the effects of simvastatin on a battery of psychological tests and inflammatory markers in healthy volunteers.

**Methods:**

Fifty-three healthy subjects were randomly assigned to 7 days of either simvastatin (*N* = 27) or sucrose-based placebo (*N* = 26) given in a double-blind fashion. Then, participants were administered questionnaires measuring subjective rates of mood and anxiety, and a battery of tasks assessing emotional processing, reward learning, and verbal memory. Blood samples for C-reactive protein were also collected.

**Results:**

Compared to placebo, participants on simvastatin showed a higher number of positively valenced intrusions in the emotional recall task (*F*_1,51_ = 4.99, *p* = 0.03), but also an increase in anxiety scores (*F*_1,51_ = 5.37, *p* = 0.02). An exploratory analysis of the females’ subgroup (*N* = 27) showed lower number of misclassifications as sad facial expression in the simvastatin arm (*F*_1,25_ = 6.60, *p* = 0.02). No further statistically significant changes could be observed on any of the other outcomes measured.

**Conclusions:**

We found limited evidence that 7-day simvastatin use in healthy volunteer induces a positive emotional bias while also being associated with an increase in anxiety, potentially reflecting the early effects of antidepressants in clinical practice. Such effect might be more evident in female subjects. Different drug dosages, treatment lengths, and sample selection need consideration in further experimental medicine and clinical studies.

**Trial registration:**

Clinicaltrials.gov: NCT04652089.

**Supplementary information:**

The online version contains supplementary material available at 10.1007/s00213-022-06156-y.

## Introduction

Statins are a class of molecules commonly prescribed to reduce peripheral cholesterol by inhibiting liver 3-hydroxy-3-methylglutaryl coenzyme A (HMG-CoA) reductases (Endo et al. 1976). In addition to their activity on lipid metabolism, statins can influence several other neurobiological, cardiometabolic, and immune systems—and especially well-established inflammatory pathways (Sirtori 2014), which appear involved in the pathophysiology of depression (De Giorgi et al. 2021a). Pooled results from small randomised controlled trials suggest that statins could effectively decrease depressive symptoms when given as add-on to antidepressants in patients suffering from clinical depression (De Giorgi et al. 2021b). Conversely, evidence from observational studies (Kessing 2021; Lee et al. 2021; Molero et al. 2020) and clinical trials (Yatham et al. 2019—see subgroup analysis) in non-clinically depressed populations is conflicting.

Treatment with conventional antidepressants leads to early positive changes in emotional processing and reward learning in both healthy volunteers and patients with depression, while drugs with depressogenic potential have opposite effects (Godlewska and Harmer 2021). Experimental manipulation of inflammatory processes has also been associated with shifts in emotional bias (Cooper et al. 2018), reward-seeking behaviour (Felger and Treadway 2016), and overall cognitive performance (Paine et al. 2015). Thus, investigating the effect of statins on these neuropsychological domains can provide valuable translational information about their potential influence on mood and cognition.

We have previously observed that 7-day treatment with atorvastatin, compared to placebo, in healthy subjects increases the processing of fearful facial expressions independently from subjective states of mood and anxiety, and the peripheral inflammatory marker, C-reactive protein (De Giorgi et al. 2021c). In line with this, an observational study has shown that atorvastatin use is associated with a higher risk of new depression diagnosis; intriguingly though, simvastatin has the opposite effect (Redlich et al. 2014). Further, clinical evidence seems to support a greater antidepressant potential for simvastatin as compared to atorvastatin (Abbasi et al. 2015; De Giorgi et al. 2021b; Molero et al. 2020), and ongoing clinical trials in depressed individuals are indeed employing simvastatin (Husain et al. 2019; Otte et al. 2020). From a psychopharmacological perspective, simvastatin differs from atorvastatin by means of being less potent at reducing peripheral cholesterol (Jones et al. 1998), but it is also considerably more lipophilic, thus more capable of crossing the blood–brain barrier and expressing its effects within the central nervous system (CNS) (McFarland et al. 2014). On these bases, the potential neuropsychological effects of simvastatin warrant further investigation.

### Aim of the study

This study is an experimental medicine trial in healthy volunteers. Its primary aim is to investigate the short-term (7 days) effect of simvastatin on a battery of emotional processing tasks that are sensitive to the early effects of typical antidepressants, such as selective serotonin inhibitors (SSRIs), on emotional bias. Changes in emotional bias in healthy participants (i.e., a group easier to recruit especially during the COVID-19 pandemic), are less affected by cognitive deficits oftentimes seen in depression, while retaining translational value for this disorder (Godlewska and Harmer 2021). Secondarily, we examine the effects of simvastatin on reward learning, verbal memory, and inflammation as measured by the inflammatory marker high-sensitivity C-reactive protein (hs-CRP). Based on existing literature proposing that statins may exert antidepressant and anti-inflammatory effects, we hypothesise that 7-day simvastatin would enhance positive bias, reward sensitivity, and cognition.

## Methods

The study was a double-blind, parallel group, randomised, gender-stratified, placebo-controlled, experimental medicine trial, approved by the University of Oxford Central University Research Ethics Committee (MS-IDREC R69606/RE001). The study protocol, which describes the methods in detail, was registered on Clinicaltrials.gov (NCT04652089).

### Sample size calculation

Based on the primary outcome of accuracy at recognising emotional facial expressions from a previous study of antidepressants in healthy volunteers (Harmer et al. 2004), we calculated that a sample size (*N*) = 38 would give 90% power to detect changes of a comparable magnitude (citalopram vs placebo effect size *F* = 0.5, see Supplementary Material, S1). G*Power v3.1.9.6 (Faul et al. 2007) software was used for this analysis.

### Participant recruitment

To account for potential difficulties in study retention due to the COVID-19 pandemic, we recruited above the number of participants estimated through the sample size calculation. Ultimately, 53 participants [both males and females, aged between 18 and 50 years, body mass index (BMI) ranging from 18 to 30] were recruited for this study. All provided full written informed consent.

Inclusion and exclusion criteria were pre-specified in the study protocol and are reported in full in the Supplementary Material, S2. In brief, volunteers were considered eligible if they were free from any current or past mental illness on the basis of their self-reported psychiatric history as well as the administration of the structured clinical interview for DSM-5 (First et al. 2016) by a study clinician. Self-reported medical and drug history were also gathered to ensure that potential participants did not suffer from any significant physical illness and were not taking any regularly prescribed medications, including any psychoactive drugs over the previous 3 months. Women who were pregnant, breastfeeding, or of child-bearing potential not using an approved contraceptive therapy were excluded. Participants who withdrew from the study were replaced as per protocol.

### Baseline procedures

Enrolled participants completed a battery of questionnaires including the Beck Depression Inventory (BDI, Beck et al. 1961), the Eysenck Personality Questionnaire (EPQ, Eysenck et al. 1985), the Positive and Negative Affect Scale (PANAS, Watson et al. 1988), the Snaith-Hamilton Pleasure Scale (SHAPS, Snaith et al. 1995), a side effect questionnaire (nausea, dizziness, dry mouth, headache, alertness, and agitation scored from 0 to 3), the State-Trait Anxiety Inventory (STAI, Spielberger et al. 1970), and the Bond-Lader Visual Analogue Scales (BL-VAS, Bond and Lader 1974). Also, a blood sample to determine serum hs-CRP levels was collected and frozen in two 1.0-mL FluidX tubes.

A randomisation code had been designed by a researcher uninvolved in the study using an online randomisation tool (https://www.sealedenvelope.com/simple-randomiser/v1/lists). Accordingly, participants were randomised to either simvastatin 20 mg [a dose used in previous clinical trials of simvastatin in depression; Gougol et al. 2015; Abbasi et al. 2015] or sucrose placebo, encapsulated in identical opaque white capsules, taken orally once daily, at night, for 7 days [based on previous evidence suggesting that the anti-inflammatory effects of statins are seen as early as 4 ± 3 days; Macin et al. 2005].

### Testing visit procedures

Following the 7 days of assigned treatment, participants returned for a testing visit. On this occasion, they reported their adherence and brought back their capsules’ bottles to verify compliance.

Participants completed some of the previously administered questionnaires (PANAS, side effect questionnaire, STAI-state, BL-VAS), and a second blood sample for hs-CRP (both the baseline and testing visit blood samples were assayed at the end of the study via automated immunoassay using the Architect c16000 analyser).

Finally, participants were administered a battery of neuropsychological tasks including the emotional test battery (ETB), the probabilistic instrumental learning task (PILT), and the auditory-verbal learning task (AVLT), described in detail in a previous similar study (De Giorgi et al. 2021c).

#### Emotional test battery

The ETB assesses emotional processing via five validated (Harmer et al. 2009) computerised tasks: the facial expression recognition task (FERT), the emotional categorisation task (ECAT), the emotional recall task (EREC), the emotional recognition memory task (EMEM), and the faces dot-probe task (FDOT).

The FERT involves the facial expressions of six emotions: anger, disgust, fear, happy, sad, surprise, and a neutral one, adapted from the Karolinska directed emotional faces set (Lundqvist et al. 1998), each expression depicted at a range of intensity levels (Young et al. 1997) and randomly displayed on the computer screen for 500 ms. Participants have to identify the presented facial expression as quickly and as accurately as possible by pressing a labelled key on the keyboard. Accuracy at recognising the correct emotion was the primary outcome measure for this study. A signal detection analysis is used to assess discriminability (*d′*, perceptual choice, a measure of sensitivity) and response bias (*β*, decisional choice, a measure of conservativeness) (Grier 1971) for this outcome. Misclassifications and mean reaction times for each facial expression are also noted.

The ECAT involves thirty positively and thirty negatively valenced words (Anderson 1968) randomly displayed on the computer screen for 500 ms. Participants have to indicate as quickly and as accurately as possible whether they would like or dislike to be referred to as one of these words. Accuracy and mean reaction times for positive and negative words are noted. The EREC is an unexpected free recall task during which participants have to write down as many self-referent words from the ECAT as possible in 4 min. Correctly (hits) and falsely recalled (false alarms) positive and negative words are noted. The EMEM involves the sixty self-referent words from the ECAT and sixty matched distractors (thirty positive, thirty negative) randomly displayed on the computer screen for 500 ms. Participants have to recognise whether these words have been previously seen (familiar) or not (novel) in the ECAT. Accuracy, false alarms and reaction times are noted.

The FDOT involves two faces, one neutral and one emotional (either happy or fearful), briefly displayed at the top and at the bottom of the computer screen and then replaced by a pair of dots with two different (vertical or horizontal) orientations. Participants have to indicate whether the dots are vertically or horizontally aligned. Mean reaction times from trials when probes are in the same position as the emotional face (congruent trials) are subtracted from mean reaction times from trials when probes are in the opposite position to the emotional face (incongruent trials) to calculate attentional vigilance scores.

#### Probabilistic instrumental learning task

The PILT, adapted from Pessiglione et al. (2006), assesses reward learning. Two pairs of symbols are displayed on the computer screen for 4000 ms: one pair is associated with win outcomes (win £0.20 or no change) and the other with loss outcomes (lose £0.20 or no change). Each symbol in the pair has reciprocal probabilities (70% or 30%) of either outcome occurring and is randomly positioned either to the left or the right of a central fixation cross. Participants began the task with £1.50 and perform sixty independent trials (thirty win trials and thirty loss trials) for two runs, receiving outcome feedback (win or lose) after each trial. Participants have to use the outcome feedback to gradually learn the symbol-outcome associations over time, to consistently choose the symbol with the high-probability win while avoiding the symbol with the high-probability loss. End total amount, amount won, amount lost, reward and lose trials, and number of choice switches (proportion of trials in which the chosen symbol is different to the symbol chosen in the previous trial within the same condition, such that the lower the proportion of switch trials, the greater the confidence the participant is thought to have had in their choices) are noted.

#### Auditory-verbal learning task

The AVLT assesses verbal memory (Rey, 1964) through a list of fifteen nouns that participants have to recall and tell to the researcher. After five repetitions of free recall, a second “interference” list of further fifteen nouns is assessed in the same manner. Then, participants have to recall the words from the first list both immediately and after a 20-min delay. Correct words, intrusions, and repetitions are noted. Finally, a list of fifty words containing all of the words from the two lists as well as distracting words is presented. Participants have to indicate whether each word belong to the first list. Correctly recalled (hits) and falsely recalled (false alarms) words are noted.

### Statistical analyses

We used the IBM SPSS v27 statistical package (IBM 2020) for all the analyses.

Participants’ demographics, clinical characteristics, and baseline questionnaires were reported descriptively. Repeated questionnaires (PANAS, side effect questionnaire, STAI-state, BL-VAS) and hs-CRP were analysed via repeated measures analysis of variance (ANOVA) with group (simvastatin versus placebo) as the between-subject factor and time (baseline versus testing visit) as the within-subject factor.

We visually checked data distributions for all neuropsychological tasks using boxplots: extreme outliers (data values that lie more than three times the interquartile range below the first quartile or above the third quartile) were excluded. The resulting data were then analysed using repeated measures ANOVA with group as the between-subject factor and emotion/valence as the within-subject factor. Significant interactions were followed up using simple main effect analyses. When assumptions of equality of variances were not fulfilled, the Greenhouse–Geisser correction was used. Partial eta squared (*η*^2^) was reported for the main significant comparisons as a measure of effect size (*η*^2^ = 0.01, small effect; *η*^2^ = 0.06, medium effect; *η*^2^ = 0.14, large effect). On the basis of potentially diverse physiopathological effects of simvastatin, we further explored the main task (FERT accuracy, misclassifications, reaction times) in specific subgroups of participants (females versus males, BMI 18–25 [normoweight] versus 26–30 [overweight], hs-CRP < 1 mg/dL versus ≥ 1 mg/dL, self-report of family history of mental disorder *positive* versus *negative*).

## Results

### Baseline measures

Participants’ demographics, clinical characteristics, and baseline questionnaires (BDI, EPQ, SHAPS, STAI-trait) are reported in Table 1. Twenty-six participants were randomised to placebo (thirteen females) and twenty-seven (fourteen females) to simvastatin.

**Table 1:** Participants’ demographics, clinical characteristics, and baseline questionnaires

		**Placebo**	**Simvastatin**
Sample size		26	27
Gender		13 F/13 M	14 F/13 M
Age		28.1 (5.7)	25.4 (3.3)
English as first language		13	20
Education	High school/college	1	3
	Undergraduate	12	13
	Postgraduate	13	11
Family history of mental disorder		11	9
Smoke/day		0.1 (0.4)	0.5 (2.0)
Alcohol units per week		4.6 (6.7)	5.5 (5.5)
Caffeinated drinks per day		1.8 (1.4)	1.6 (1.5)
BMI		22.8 (3.0)	22.3 (2.6)
BDI		2.8 (3.0)	2.7 (4.2)
EPQ	Neuroticism/stability	8.8 (3.9)	8.1 (4.3)
	Psychoticism/socialisation	2.3 (0.3)	3.4 (3.2)
	Extroversion/introversion	12.7 (5.1)	14.4 (3.9)
	Lie/social desirability	10.8 (4.2)	10.00 (4.2)
SHAPS		1.3 (1.9)	0.4 (0.6)
STAI-t		34.6 (8.3)	34.6 (7.3)

Values are means with (standard deviations).

*BDI*, Beck Depression Inventory; *BMI*, body mass index; *EPQ*, Eysenck Personality Questionnaire; *SHAPS*, Snaith-Hamilton Pleasure Scale; *STAI-t*, State-Trait Anxiety Inventory-Trait.

### Testing visit measures

Participants’ repeated questionnaires (PANAS, STAI-state, side effect questionnaire) and hs-CRP are shown in Table 2.

**Table 2:** Participants’ repeated questionnaires and hs-CRP

	**Placebo**		**Simvastatin**		**Visit** (*p*)	**Group × visit** (*p*)
	Screening visit	Research visit	Screening visit	Research visit		
hs-CRP	1.0 (0.7)	1.1 (0.8)	0.7 (0.4)	0.8 (0.6)	** > **0.20	> 0.20
PANAS-positive	31.9 (7.2)	32.5 (7.8)	30.9 (7.0)	31.9 (6.4)	> 0.20	> 0.20
PANAS-negative	12.4 (2.5)	12.9 (2.9)	13.0 (3.0)	14.3 (4.6)	> 0.20	> 0.20
Side effects	0.4 (0.6)	0.5 (0.8)	0.5 (0.9)	0.7 (0.2)	> 0.20	> 0.20
STAI-s	31.4 (9.0)	31.7 (8.5)	29.9 (6.4)	35.6 (9.7)	0.01*	0.02*

Values are means with standard deviations. An asterisk (*) highlights a statistically significant difference between the simvastatin and placebo groups.

*hs-CRP*, high-sensitivity C-reactive protein; *PANAS*, Positive and Negative Affective Schedule; *Side-effects*, side effects questionnaire; *STAI-s*, State-Trait Anxiety Inventory-State.

**Fig. 1:**
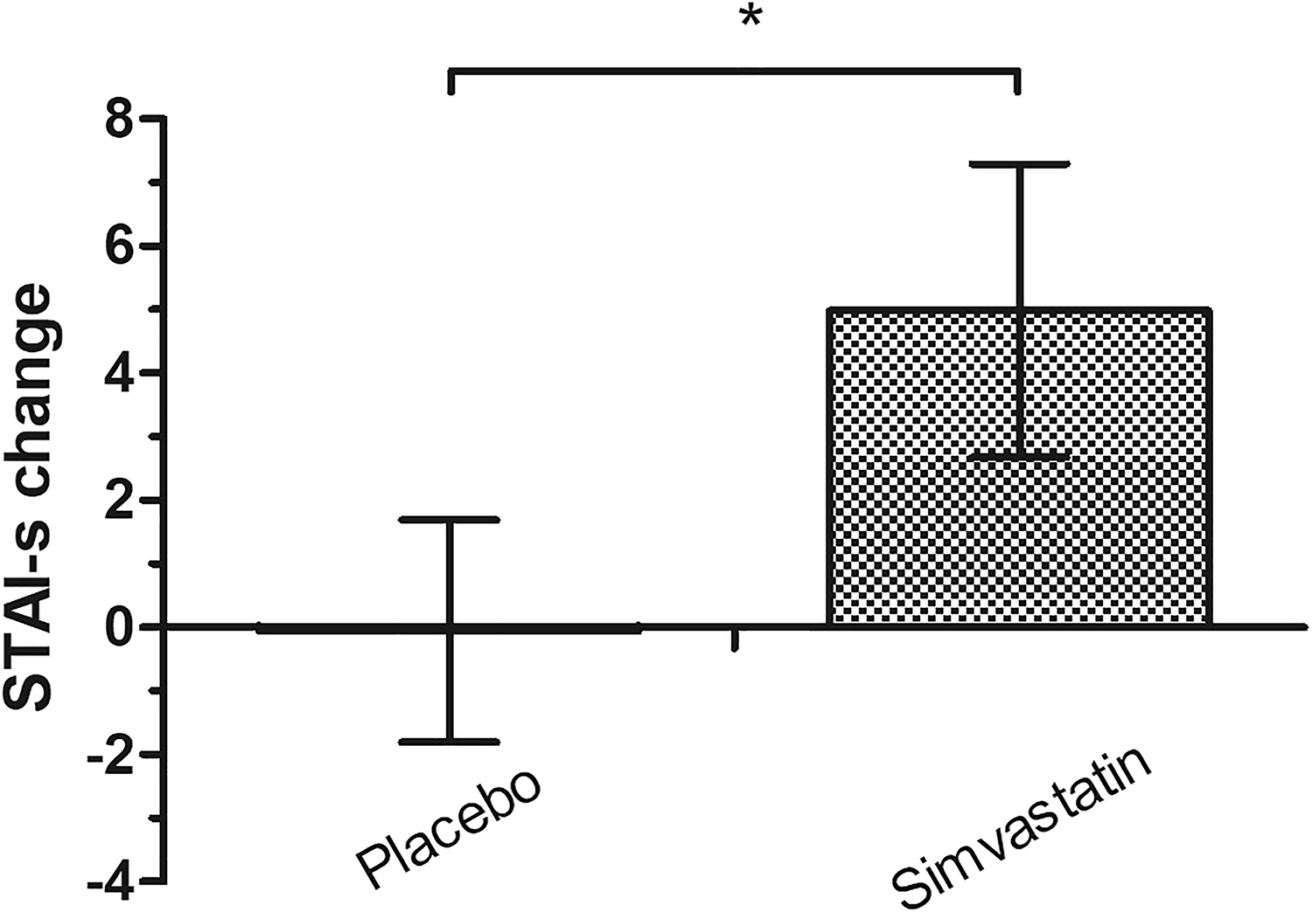
Effect of simvastatin on the State-Trait Anxiety Inventory-State (STAI-s) score. Values are mean differences between visits ± standard error of the mean bars, and an asterisk (*) represents a statistically significant difference between the simvastatin (grey) and placebo (white) groups

The STAI-state showed a main effect of time (*F*_1,51_ = 6.91, *p* = 0.01) and a group-time interaction (*F*_1,51_ = 5.37, *p* = 0.02) due to higher scores at the testing visit for the simvastatin group (Fig. 1). The other questionnaires and hs-CRP showed no effect of time or group-time interaction (*F*_s_ < 2.97, *p*_s_ > 0.20).

For the BL-VAS, several items showed a statistically significant main effect of time, but no significant group-time interaction was observed (*F*_s_ < 4.10, *p*_s_ > 0.20) (see Supplementary Material, S3).

The descriptive statistics for all neuropsychological outcomes, including the number of outliers excluded, are reported in the Supplementary Material, S4. The very low number of outliers did not compromise our conservative sample size for all tasks.

### Emotional test battery results

### FERT

For facial expression recognition, there was no significant group-emotion interaction for any of the outcome measures (*F*_s_ < 1.40, *p*_s_ > 0.20) (see Supplementary Material, S5).

When we assessed the effect of gender on the FERT (see Supplementary Material, S4 and Supplementary Material, S5), we observed a group-gender-emotion interaction for misclassifications that approached statistical significance (*F*_6,43_ = 2.50, *p* = 0.07). This was driven by a reduced tendency to mislabel other facial expressions as sad in the simvastatin group amongst female participants (*F*_1,48_ = 6.91, *p* = 0.01, *η*^2^ = 0.13). We therefore explored this effect within the subgroup of females (placebo *N* = 13, simvastatin *N* = 14). A group-emotion interaction for misclassifications was confirmed (*F*_6,20_ = 2.57, *p* = 0.05), which again was due to a lower number of misclassifications as sad facial expression for the simvastatin group (*F*_1,25_ = 6.60, *p* = 0.02, *η*^2^ = 0.21). A further analysis clarified that females on simvastatin misclassified fewer positive facial expressions, when compared to negative emotions, as sad facial expressions (*F*_1,25_ = 5.79, *p* = 0.02, *η*^2^ = 0.19). None of the other pre-specified variables (BMI, hs-CRP, family history of mental disorder—see Supplementary Material, S4) had any effect on this task’s outcomes (*F*_s_ < 3.34, *p*_s_ > 0.20).

### ECAT, EREC, and EMEM

**Fig. 2:**
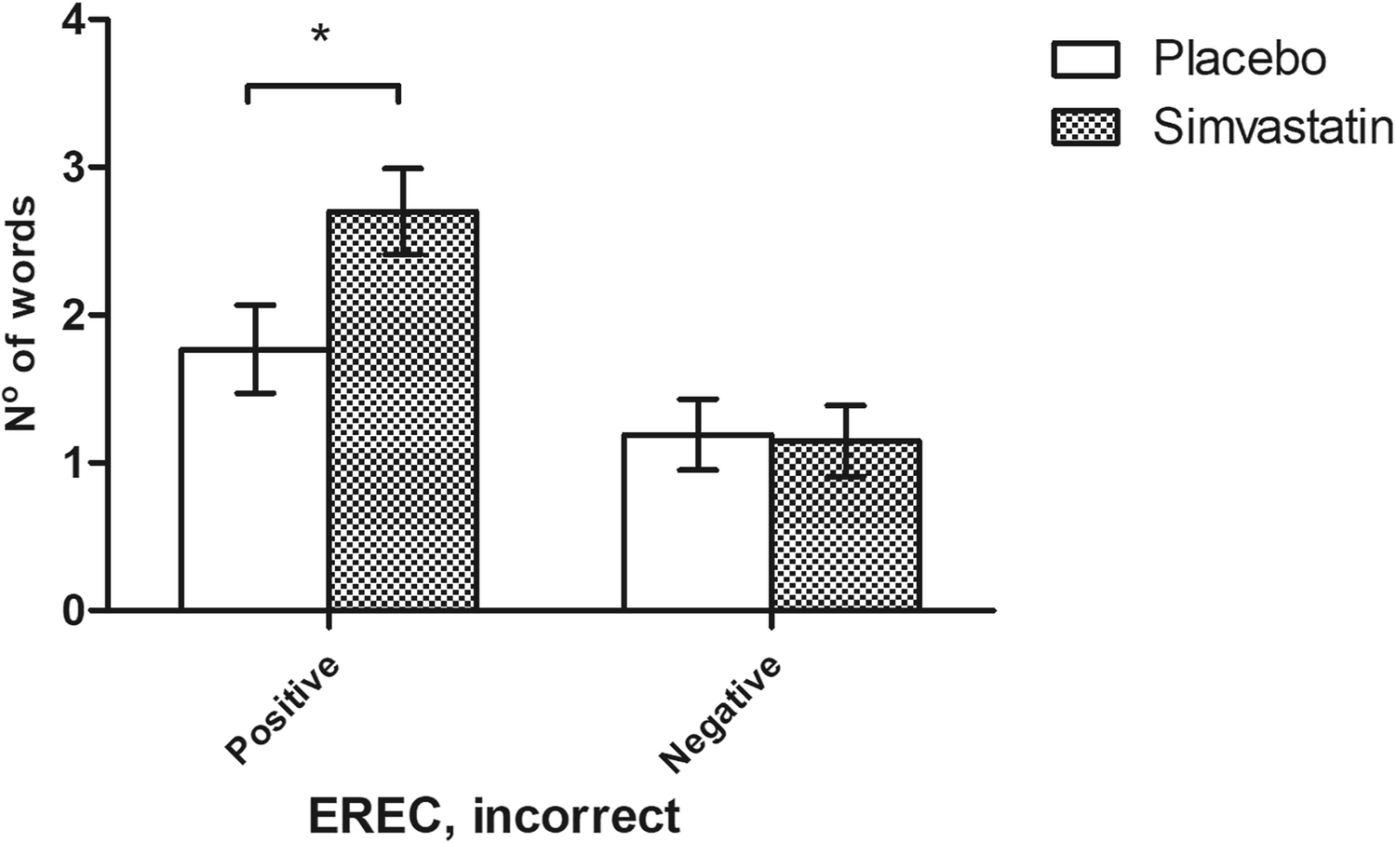
Effect of simvastatin on emotional recall (EREC), false alarms. Values are means ± standard errors of the mean bars, and an asterisk (*) represents a statistically significant difference between the simvastatin (grey) and placebo (white) groups

For emotional recall, we observed a group-valence interaction (*F*_1,51_ = 4.86, *p* = 0.03), where individuals on simvastatin recalled more positive intrusions compared to placebo (*F*_1,51_ = 4.99, *p* = 0.03, *η*^2^ = 0.09). No effect on negatively valenced words was identified (*F*_1,51_ = 0.02, *p* > 0.20) (Fig. 2). There were no further significant group-valence interactions for any of the other ECAT, EREC, and EMEM outcomes measured (*F*_s_ < 2.90, *p*_s_ > 0.20).

### FDOT

There was no significant group-emotion interaction for attentional vigilance towards happy or fearful faces on any of the FDOT outcomes (*F*_s_ < 0.97, *p*_s_ > 0.20).

### Probabilistic instrumental learning task results

For reward learning, there was no significant effect of simvastatin on any of the outcome measures (*F*_s_ < 1.43, *p*_s_ > 0.20). The learning curves are reported in the Supplementary Material, S6.

#### Auditory-verbal learning task results

For verbal memory, no significant differences between the simvastatin and placebo groups were identified for any of the outcomes measured (*F*_s_ < 2.70, *p*_s_ > 0.20).

## Discussion

In this experimental medicine trial in healthy volunteers, we investigated the effects of the highly lipophilic, widely used simvastatin on a battery of tests conducted under laboratory conditions. Seven-day simvastatin 20 mg treatment, compared to placebo, did not lead to any statistically significant changes on the majority of the emotional processing, reward learning, and verbal memory outcomes measured, nor it did for most of the questionnaires administered and hs-CRP levels. However, we found some evidence that simvastatin may induce a positive bias in emotional processing as evidenced by the increased number of positively valenced intrusions in the EREC task when compared to placebo, and reduced labelling of faces as sad in females taking simvastatin. At the same time, simvastatin use was associated with higher anxiety levels as measured by the STAI-s questionnaire. Taken together, these complex findings require further interpretation.

### Effects of simvastatin on emotional processing and anxiety scores

A simple explanation for our results would be that simvastatin does not affect any of the cognitive domains assessed, and that the only effect we observed on emotional recall was coincidental. However, this conclusion contrasts with growing evidence from clinical studies that statins have an antidepressant potential (De Giorgi et al. 2021a). Early changes in emotional processing produced by antidepressants can predict later effects on clinical mood states (Godlewska and Harmer 2021), but it is possible that statins’ putative antidepressant effects are related to physiopathological pathways that are not well-captured by the neuropsychological tasks employed here.

An alternative explanation for the modest effect seen on emotional processing is that higher doses or longer periods of treatment are needed for more indicative data. Simvastatin is commonly used at doses between 10 and 40 mg for several years and, although dosage can be increased to 80 mg, its potency remains lower than atorvastatin and rosuvastatin (Chou et al. 2016). Studies investigating whether there is any dose–response relationship between simvastatin use, emotional processing, and its several effects on lipids and inflammation could clarify this matter.

An indication that simvastatin may have antidepressant-like effects at higher doses or longer administration periods may lie in the observed dissociable effects between worsened subjective anxiety scores and mildly improved emotional processing. Comparable results were previously obtained in another experimental medicine trial where a single oral dose of the serotonergic and noradrenergic reuptake inhibitor (SNRI), duloxetine, led to an increased recall of false alarms for positive personality characteristics in the EREC, while also worsening anxiety and other subjective mood states (Harmer et al. 2008). Intriguingly, this pattern seems to reflect routine clinical experience with most antidepressant medications: an initial exacerbation of anxiety followed by antidepressant and anxiolytic effects (Harmer et al. 2011)—a reversal of action likely involving desensitisation of 5HT_2c_ receptors (Deakin and Graeff 1991). Hence in our study, 7-day simvastatin might have caused the earlier, paradoxical (and in this case, captured) increase in anxiety before showing any antidepressant-like effect (as suggested by a more positive outcome on the EREC). Compared to conventional antidepressants, the intricate pharmacological actions of statins (Walker et al. 2021) could require longer administration before showing the expected positive effects on emotional processing—and eventually on scales of mood and anxiety, as suggested by clinical studies (De Giorgi et al. 2021a). Evidence from experimental medicine trials of statins with longer periods of treatment (NCT04973800) or employing more sensitive methodologies, such as neural measures of emotional processing via fMRI (Godlewska and Harmer 2021), could shed some light on this issue.

### Potential psychopharmacological pathways involved in simvastatin’s action

From a pharmacological perspective, simvastatin use seemed more associated with an increase in positive bias in emotional memory, similar to noradrenergic antidepressants, rather than a decrease in negative bias and emotional expression recognition that is usually seen with serotonergic drugs (Pringle et al. 2013). This observation was unexpected, though simvastatin’s effects on noradrenergic circuits may occur indirectly, for example, via interconnected anti-inflammatory pathways (Walker et al. 2021). Current evidence from both animal and human studies has thus far mainly confirmed an effect of statins on central serotonin and dopamine turnover, amongst monoamines (De Giorgi et al. 2021a).

Another important consideration concerns the apparent lack of effect of simvastatin on reward learning, a dopamine-dependent function (Pessiglione et al. 2006), as measured by the PILT. Altered dopamine neurotransmission has been particularly associated with inflammation and implicated in an inflammatory phenotype of depression characterised by significant anhedonia (Miller et al. 2017). Therefore, one could expect that the anti-inflammatory properties of simvastatin should have affected reward learning via their activity on dopaminergic pathways. This negative finding may be due to our sample of healthy volunteers having overall low baseline inflammatory levels, as measured by CRP—a study limitation further discussed below. Another possibility is that, once again, more than 7 days of simvastatin’s administration might have been required to see an effect on the PILT, as suggested by a previous study of the norepinephrine-dopamine reuptake inhibitor (NDRI), bupropion, whose action on reward learning could be detected at 2 (negative effect) and 6 (positive effect) weeks of treatment (Walsh et al. 2018).

### Simvastatin versus atorvastatin effects

Our findings also appear surprising when compared to a parallel experimental medicine trial where 7-day atorvastatin was shown to increase the processing of fearful faces, normally linked to anxiety states (Mogg and Bradley 2002), in the absence of any changes for mood and anxiety questionnaire scores (De Giorgi et al. 2021c). On the assumption that a drug’s ability to penetrate the CNS is important for expressing an activity on emotional processing, one would expect to see these same results replicated—perhaps even to a greater extent, since simvastatin is more lipophilic than atorvastatin (McFarland et al. 2014). Consistent with this, we observed an increase in anxiety state on the STAI-s questionnaire; however, typically more sensitive measures of fear processing (FERT, FDOT) were not affected, and indeed, we observed a positive effect on emotional recall. Complex CNS and peripheral interactions between simvastatin and inflammation, lipid metabolism, neurotransmitters, and perhaps other less-known factors (De Giorgi et al. 2021a) likely underlie these composite outcomes. Another experimental medicine trial with the highly hydrophilic molecule, rosuvastatin, could elucidate whether a drug’s direct brain penetrance, as compared to other peripheral actions with downstream CNS effects, is necessary for influencing the neuropsychological tasks employed. These pharmacological issues can have a meaningful translational value, as in the context of certain evidence suggesting that peripheral (rather than central) inflammation is key in the inflammatory hypothesis of depression (Bullmore 2018). Nevertheless, it should be noted that clinical trials have been unable to confirm an antidepressant effect for rosuvastatin (Berk et al. 2020), whereas a step-wise increase in antidepressant activity according to statin’s lipophilicity has been suggested (De Giorgi et al. 2021b).

### Considering “precision” in experimental medicine trials

Experimental medicine trials conducted in healthy subjects are an essential proof-of-concept for the cognitive neuropsychological model of depression and antidepressant action (Godlewska and Harmer 2021), and can support go/no-go decisions for the development or repurposing of new antidepressant drugs (Harmer et al. 2011). However, in the case of statins or similar medications with such complex pharmacological activity on several bodily systems, further experimental medicine trials should focus on specific subpopulations that are prone to show a neuropsychological response to such interventions (Cotter and Barnett 2018), similarly to what precision psychiatry suggests for clinical studies (Fernandes et al. 2020). For example, clinical trials of anti-inflammatory medications for depressive disorder should pre-select subgroups of patients with raised inflammatory markers, such as peripheral CRP, as they are more likely to benefit from these treatments (Chamberlain et al. 2019).

For statins, participants’ baseline lipid and inflammatory profile should be taken into account when interpreting their effects on both neuropsychological markers and depressive scales. Statins’ chief activity is enacted on lipid metabolism and inflammation (Jain and Ridker 2005); both have been linked to depression (Miller et al. 2017; Walther et al. 2018) as well as the interaction between these two elements have (van Diepen et al. 2013). In line with these observations, a recent study in healthy volunteers has shown that minocycline, an antibiotic with putative antidepressant action, reduces negative affective bias and CRP levels while also increasing cholesterol (Chan et al. 2020). Our current experimental medicine trial did not find any significant change in hs-CRP between the simvastatin and placebo groups, and no interaction effect was detected between its levels and our main neuropsychological task (FERT). However, hs-CRP levels were generally low (i.e., < 1 mg/dL) for both groups in our sample of healthy participants, thus limiting the validity of such finding. Moreover, we did not measure pre- and post-intervention cholesterol levels. Taken together, these are avenues warranting consideration for future studies on statins—whether clinical or translational.

Participants’ age and overall health status are further key issues—it is noteworthy that the current study was conducted in young, healthy volunteers. It has been speculated that older people might be more likely to experience a depressogenic reaction to statins as they would have less ability to compensate for the wide range of neurobiological changes produced by statin treatment (Kim et al. 2019). Nevertheless, a large observational study highlighted an association between advancing age and lower odds of depression for statin users (Redlich et al. 2014), possibly due to elderly people benefitting more from this intervention because of age-related higher baseline inflammation (Ferrucci and Fabbri, 2018). For similar reasons, people who are at-risk of developing depressive episodes through factors such as obesity (Capuron et al. 2017) or positive family history of mood disorders (Monroe et al. 2014) might be important participants for further statin studies. However, our trial could not identify any interaction between statin use, BMI, or psychiatric family history, and the FERT, perhaps owing to small sample size.

In this context, our exploratory analysis on the subgroup of 27 female participants (i.e., lower than the number required to see an effect on the FERT according to our sample size calculation) highlights intriguing, though preliminary, findings. There is evidence of sex differences at the interface between immunity and depression (Rainville et al. 2018), with inflammation seeming to be a more conspicuous driver of depressive symptomatology in females compared to males (Köhler-Forsberg et al. 2017). Consistent with this, we observed a more positive effect of simvastatin in females as evidenced by fewer misclassifications of facial expressions as sad, and indeed specifically of positive facial expressions as sad—another sign of induction of positive bias. Once again, this finding must be interpreted with caution because of its post hoc nature on a small sample. However, it raises the possibility that these results could be replicated more convincingly in further studies including only female subjects, or with higher sample sizes and a pre-specified analysis plan focussing on females.

### Limitations

In addition to the study limitations described above, it should be noted that we conducted numerous analyses, other than accuracy for facial expression recognition, that were not corrected for multiple comparisons because of small sample size, thus increasing the chances of false-positive results. Conversely, our sample size had been calculated on the main outcome of accuracy at recognising emotional facial expressions; therefore, it is possible that we could not find any statistically significant effect of simvastatin on other outcome measures because our trial had not been powered to detect that.

### Conclusions

Our experimental medicine trial in healthy volunteers found that 7-day simvastatin administration, compared to placebo, was associated with an increase in subjective anxiety score while possibly inducing a positive bias in the recall of emotional information. No further effects on emotional processing, reward learning, and verbal memory could be detected. These findings do not have any current clinical application, but can inform future experimental medicine and clinical trials on the effects of statins in depression and anxiety. A finer tailoring of the administered dose, length of treatment, molecule of choice, and target population might lead to more significative results. Together with evidence from other pre-clinical and clinical studies, we cautiously suggest that further studies should focus on the highly lipophilic simvastatin, administered at doses ≥ 20 mg/day for ≥ 7 days, in people with depression and baseline altered lipid and inflammatory profiles.

## Supplementary information

Below is the link to the electronic supplementary material.

Supplementary file1 (DOCX 3131 KB)

## References

[CR1] Abbasi SH, Mohammadinejad P, Shahmansouri N, Salehiomran A, Beglar AA, Zeinoddini A, Forghani S, Akhondzadeh S (2015). Simvastatin versus atorvastatin for improving mild to moderate depression in post-coronary artery bypass graft patients: a double-blind, placebo-controlled, randomized trial. J Affect Disord.

[CR2] Anderson NH (1968). Likableness ratings of 555 personality-trait words. J Pers Soc Psychol.

[CR3] Beck AT, Ward CH, Mendelson M, Mock J, Erbaugh J (1961). An inventory for measuring depression. Arch Gen Psychiatry.

[CR4] Berk M, Mohebbi M, Dean OM, Cotton SM, Chanen AM, Dodd S, Ratheesh A, Amminger GP, Phelan M, Weller A, Mackinnon A, Giorlando F, Baird S, Incerti L, Brodie RE, Ferguson NO, Rice S, Schäfer MR, Mullen E, Hetrick S, Kerr M, Harrigan SM, Quinn AL, Mazza C, McGorry P, Davey CG (2020). Youth Depression Alleviation with Anti-inflammatory Agents (YoDA-A): a randomised clinical trial of rosuvastatin and aspirin. BMC Med.

[CR5] Bond A, Lader M (1974) The use of analogue scales in rating subjective feelings. Br J Med Psychol 47

[CR6] Bullmore E (2018). The art of medicine: inflamed depression. Lancet.

[CR7] Capuron N, Lasselin J, Castanon N (2017). Role of adiposity-driven inflammation in depressive morbidity. Neuropsychopharmacol.

[CR8] Chamberlain SR, Cavanagh J, de Boer P, Mondelli V, Jones DNC, DrevetsWC Cowen PJ, Harrison NA, Pointon L, Pariante CM, Bullmore ET (2019). Treatment-resistant depression andperipheral C-reactive protein. Br J Psychiatry.

[CR9] Chan SY, Capitão L, Probert F, Klinge C, Hoeckner S, Harmer CJ, Cowen PJ, Anthony DC, Burnet PWJ (2020). A single administration of the antibiotic, minocycline, reduces fear processing and improves implicit learning in healthy volunteers: analysis of the serum metabolome. Transl Psychiatry.

[CR10] Chou R, Dana T, Blazina I, Daeges M, Bougatsos C, Grusing S, Jeanne TL (2016) Statin use for the prevention of cardiovascular disease in adults: A systematic review for the U.S. preventive services task force. Agency for Healthcare Research and Quality (US) 27905702

[CR11] Cooper CM, Godlewska B, Sharpley AL, Barnes E, Cowen PJ, Harmer CJ (2018). Interferon-α induces negative biases in emotional processing in patients with hepatitis C virus infection: a preliminary study. Psychol Med.

[CR12] Cotter J, Barnett JH (2018). Using affective cognition to enhance precision psychiatry. Front Psychiatry.

[CR13] Deakin JFW, Graeff FG (1991). 5-HT and mechanisms of defence. J Psychopharmacol.

[CR14] De Giorgi R, De Crescenzo F, Rizzo Pesci N, Martens M, Howard W, Cowen PJ, Harmer CJ (2021). Statins for major depressive disorder: a systematic review and meta-analysis of randomized controlled trials. PlosONE.

[CR15] De Giorgi R, Martens M, Rizzo Pesci N, Cowen PJ, Harmer CJ (2021). The effects of atorvastatin on emotional processing, reward learning, verbal memory and inflammation in healthy volunteers: an experimental medicine study. J Psychopharmacol.

[CR16] van Diepen JA, Berbée JF, Havekes LM, Rensen PC (2013). Interactions between inflammation and lipid metabolism: relevance for efficacy of anti-inflammatory drugs in the treatment of atherosclerosis. Atherosclerosis.

[CR17] Endo A, Kuroda M, Tanzawa K (1976). Competitive inhibition of 3-hydroxy-3-methylglutaryl coenzyme A reductase by ML-236A and ML-236B fungal metabolites, having hypocholesterolemic activity. FEBS Lett.

[CR18] Eysenck SBG, Eysenck HJ, Barrett P (1985). A revised version of the psychoticism scale. Personal Individ Differ.

[CR19] Faul F, Erdfelder E, Lang AG, Buchner A (2007). G*Power 3: a flexible statistical power analysis program for the social, behavioral, and biomedical sciences. Behav Res Methods.

[CR20] Felger J, Treadway M (2016). Inflammation effects on motivation and motor activity: role of dopamine. Neuropsychopharmacol.

[CR21] Fernandes BS, Borgwardt S, Carvalho AF, Steiner J (2020). Editorial: back to the future: on the road towards precision psychiatry. Front Psychiatry.

[CR22] Ferrucci L, Fabbri E (2018). Inflammageing: chronic inflammation in ageing, cardiovascular disease, and frailty. Nat Rev Cardiol.

[CR23] First MB, Williams JBW, Karg RS, Spitzer RL (2016) User’s guide for the SCID-5-CV Structured Clinical Interview for DSM-5® disorders: Clinical version. Am Psychiatr Publ, Inc

[CR24] De Giorgi R, Rizzo Pesci N, Quinton A, De Crescenzo F, Cowen PJ, Harmer CJ (2021). Statins in depression: an evidence-based overview of mechanisms and clinical studies. Front Psychiatry.

[CR25] Gougol A, Zareh-Mohammadi N, Raheb S, Farokhnia M, Salimi S, Iranpour N, Yekehtaz H, Akhondzadeh S (2015). Simvastatin as an adjuvant therapy to fluoxetine in patientswith moderate to severe major depression: A double-blind placebo-controlled trial. J Psychopharmacol.

[CR26] Godlewska BR, Harmer CJ (2021). Cognitive neuropsychological theory of antidepressant action: a modern-day approach to depression and its treatment. Psychopharmacol (Berl).

[CR27] Grier JB (1971). Nonparametric indexes for sensitivity and bias: computing formulas. Psychol Bull.

[CR28] Harmer CJ, Shelley NC, Cowen PJ, Goodwin GM (2004). Increased positive versus negative affective perception and memory in healthy volunteers following selective serotonin and norepinephrine reuptake inhibition. Am J Psychiatry.

[CR29] Harmer CJ, Heinzen J, O'Sullivan U, Ayres RA, Cowen PJ (2008). Dissociable effects of acute antidepressant drug administration on subjective and emotional processing measures in healthy volunteers. Psychopharmacol (Berl).

[CR30] Harmer CJ, O'Sullivan U, Favaron E, Massey-Chase R, Ayres R, Reinecke A, Goodwin GM, Cowen PJ (2009). Effect of acute antidepressant administration on negative affective bias in depressed patients. Am J Psychiatry.

[CR31] Harmer CJ, Cowen PJ, Goodwin GM (2011). Efficacy markers in depression. J Psychopharmacol.

[CR32] Husain MI, Chaudhry IB, Khoso AB, Husain MO, Rahman RR, Hamirani MM, Hodsoll J, Carvalho AF, Husain N, Young AH (2019). Adjunctive simvastatin for treatment-resistant depression: study protocol of a 12-week randomised controlled trial. BJPsych Open.

[CR33] IBM Corp. Released 2020 (2020). IBM SPSS Statistics for Macintosh, Version 27.0. Armonk, NY: IBM Corp

[CR34] Jain MK, Ridker PM (2005). Anti-inflammatory effects of statins: clinical evidence and basic mechanisms. Nat Rev Drug Discov.

[CR35] Jones P, Kafonek S, Laurora I, Hunninghake D (1998) Comparative dose efficacy study of atorvastatin versus simvastatin, pravastatin, lovastatin, and fluvastatin in patients with hypercholesterolemia (the CURVES study). Am J Cardiol 81(5):582–7.: 10.1016/s0002-9149(97)00965-x. Erratum in: Am J Cardiol 82(1):128 10.1016/s0002-9149(97)00965-x 9514454

[CR36] Kessing LV (2021) Incomplete systematic review and meta-analysis on statin use and depression risk - a commentary. J Affect Disord 295:215.: 10.1016/j.jad.2021.08.038 10.1016/j.jad.2021.08.038 34481149

[CR37] Kim SW, Kang HJ, Jhon M, Kim JW, Lee JY, Walker AJ, Agustini B, Kim JM, Berk M (2019) Statins and inflammation: new therapeutic opportunities in psychiatry. Front Psychiatry 10:103.: 10.3389/fpsyt.2019.00103 10.3389/fpsyt.2019.00103 PMC6413672 30890971

[CR38] Köhler-Forsberg O, Buttenschøn HN, Tansey KE, Maier W, Hauser J, Dernovsek MZ, Henigsberg N, Souery D, Farmer A, Rietschel M, McGuffin P, Aitchison KJ, Uher R, Mors O (2017). Association between C-reactive protein (CRP) with depression symptom severity and specific depressive symptoms in major depression. Brain Behav Immun.

[CR39] Lee MC, Peng TR, Lee CH, Wang JY, Lee JA, Chen SM, Shiang JC (2021) Statin use and depression risk: a systematic review and meta-analysis. J Affect Disord 282:308–315.: 10.1016/j.jad.2020.12.164 10.1016/j.jad.2020.12.164 33421857

[CR40] Lundqvist ED, Flykt A, Öhman A (1998) The Karolinska Directed Emotional Faces - KDEF, CD ROM from Department of Clinical Neuroscience, Psychology section, Karolinska Institutet, ISBN 91–630–7164–9

[CR41] Macin SM, Perna ER, Farías EF, Franciosi V, Cialzeta JR, Brizuela M, Medina F, Tajer C, Doval H, Badaracco R (2005). Atorvastatin has an important acute anti-inflammatory effect in patients with acute coronary syndrome: results of a randomized, double-blind, placebo-controlled study. Am Heart J.

[CR42] McFarland AJ, Anoopkumar-Dukie S, Arora DS, Grant GD, McDermott CM, Perkins AV, Davey AK (2014). Molecular mechanisms underlying the effects of statins in the central nervous system. Int J Mol Sci.

[CR43] Miller AH, Haroon E, Felger JC (2017). Therapeutic implications of brain-immune interactions: treatment in translation. Neuropsychopharmacol.

[CR44] Mogg K, Bradley BP (2002) Selective orienting of attention to masked threat faces in social anxiety. Behav Res 40, 1403– 1414.:10.1016/S0005- 7967(02)00017–7 10.1016/s0005-7967(02)00017-7 12457635

[CR45] Molero Y, Cipriani A, Larsson H, Lichtenstein P, D'Onofrio BM, Fazel S (2020). Associations between statin use and suicidality, depression, anxiety, and seizures: a Swedish total-population cohort study. Lancet Psychiatry.

[CR46] Monroe SM, Slavich GM, Gotlib IH (2014) Life stress and family history for depression: the moderating role of past depressive episodes. J Psychiatr Res 49:90–5.: 10.1016/j.jpsychires.2013.11.005 10.1016/j.jpsychires.2013.11.005 PMC3918432 24308926

[CR47] Otte C, Chae WR, Nowacki J, et al (2020) Simvastatin add-on to escitalopram in patients with comorbid obesity and major depression (SIMCODE): study protocol of a multicentre, randomised, double-blind, placebo-controlled trial. BMJ Open 2020;10:e040119.:https://doi.org/10.1136/bmjopen-2020–040119 10.1136/bmjopen-2020-040119 PMC7709515 33262189

[CR48] Paine NJ, Bosch JA, Ring C, Drayson MT, Veldhuijzen van Zanten JJ (2015). Induced mild systemic inflammation is associated with impaired ability to improve cognitive task performance by practice. Psychophysiol.

[CR49] Pessiglione M, Seymour B, Flandin G, Dolan RJ, Frith CD (2006). Dopamine-dependent prediction errors underpin reward-seeking behaviour in humans. Nature.

[CR50] Pringle A, McCabe C, Cowen PJ, Harmer CJ (2013) Antidepressant treatment and emotional processing: can we dissociate the roles of serotonin and noradrenaline? J Psychopharmacol 27(8):719–31.: 10.1177/0269881112474523. Erratum in: J Psychopharmacol. 27(10):964 10.1177/0269881112474523 23392757

[CR51] Rainville JR, Tsyglakova M, Hodes GE (2018) Deciphering sex differences in the immune system and depression. Front Neuroendocrinol 50:67–90.: 10.1016/j.yfrne.2017.12.004 10.1016/j.yfrne.2017.12.004 29288680

[CR52] Redlich C, Berk M, Williams LJ, Sundquist J, Sundquist K, Li X (2014) Statin use and risk of depression: a Swedish national cohort study. BMC Psychiatry 14:348.: 10.1186/s12888-014-0348-y 10.1186/s12888-014-0348-y PMC4266881 25471121

[CR53] Rey A (1964). L'examen clinique en psychologic [The clinical examination in psychology].

[CR54] Sirtori CR (2014). The pharmacology of statins. Pharmacol Res.

[CR55] Snaith RP, Hamilton M, Morley S, Humayan A, Hargreaves D, Trigwell P (1995). A scale for the assessment of hedonic tone the Snaith-Hamilton Pleasure Scale. Br J Psychiatry.

[CR56] Spielberger CD, Gorsuch RL, Lushene RD (1970). STAI manual.

[CR57] Walker AJ, Kim Y, Borissiouk I, Rehder R, Dodd S, Morris G, Nierenberg AA, Maes M, Fernandes BS, Dean OM, Williams LJ, Eyre HA, Kim SW, Zoungas S, Carvalho AF, Berk M (2021) Statins: neurobiological underpinnings and mechanisms in mood disorders. Neurosci Biobehav Rev 128:693–708.: 10.1016/j.neubiorev.2021.07.012 10.1016/j.neubiorev.2021.07.012 34265321

[CR58] Walsh AEL, Browning M, Drevets WC, Furey M, Harmer CJ (2018). Dissociable temporal effects of bupropion on behavioural measures of emotional and reward processing in depression. Philos Trans R Soc Lond B Biol Sci.

[CR59] Walther A, Cannistraci CV, Simons K, Durán C, Gerl MJ, Wehrli S, Kirschbaum C (2018) Lipidomics in major depressive disorder. Front Psychiatry 9:459.: 10.3389/fpsyt.2018.00459 10.3389/fpsyt.2018.00459 PMC6196281 30374314

[CR60] Watson D, Clarke LA, Tellegen A (1988). Development and validation of brief measures of positive and negative affect: the positive and negative affect schedule scales. J Pers Soc Psychol.

[CR61] Yatham MS, Yatham KS, Ravindran AV, Sullivan F (2019). Do statins have an effect on depressive symptoms? A systematic review and meta-analysis. J Affect Disord.

[CR62] Young AW, Rowland D, Calder AJ, Etcoff NL, Seth A, Perrett DI (1997). Facial expression megamix: tests of dimensional and category accounts of emotion recognition. Cognition.

