# Supplementary material for: An experimental medicine study of the effects of simvastatin on emotional processing, reward learning, verbal memory, and inflammation in healthy volunteers
Source: Psychopharmacology (Berl). 2022 May 5;239(8):2635–45. doi: 10.1007/s00213-022-06156-y (PMC9069418; doi:10.1007/s00213-022-06156-y)
Supplement: Supplementary file 1 — Supplementary file1 (DOCX 3131 KB) [file 213_2022_6156_MOESM1_ESM.docx]

**SUPPLEMENTARY MATERIAL**

##### S1 – Sample size calculation


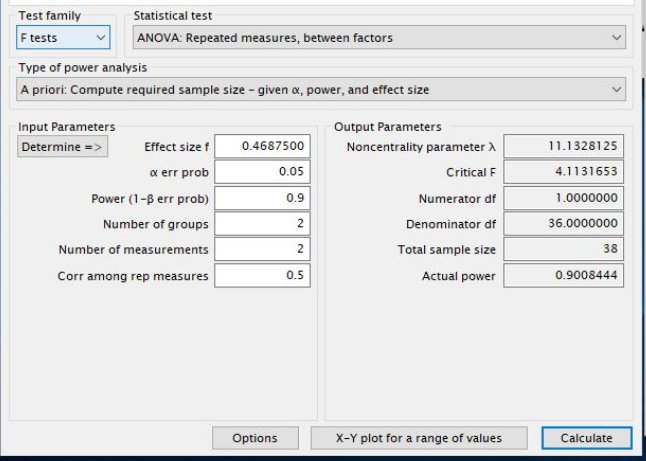


##### S2 – Inclusion/exclusion criteria

Inclusion criteria

- Male or female
- Aged 18-50 years
- Sufficiently fluent English to understand and complete the tasks
- Body Mass Index in the range of 18-30
- Participant is willing and able to give informed consent for participation in the study
- Not currently taking any regular medications (except the contraceptive pill)

Exclusion criteria

- Currently any regular medications (except the contraceptive pill)
- History or current significant psychiatric illness
- Current alcohol or substance misuse disorder
- History or current significant hepatic disease
- History or current significant neurological condition (e.g., epilepsy)
- History of haemorrhagic stroke or lacunar infarct
- Known hyperglycaemia/pre-diabetes
- Known hypersensitivity to the study drug (i.e., simvastatin) or sucrose
- Pregnant, breast feeding, women of child-bearing potential not using appropriate contraceptive measures
- Participation in a study that uses the same or similar computer tasks as those used in the present study
- Participation in a study that involves the use of a medication within the last three months

##### S3 – Participants’ repeated Bond-Lader Visual Analog Scale (BL-VAS)

|  | **Placebo** | | **Simvastatin** | | **Visit** | | **Group x Visit** | |
| --- | --- | --- | --- | --- | --- | --- | --- | --- |
|  | *Screening visit* | *Research visit* | *Screening visit* | *Research visit* | *F* | *p* | *F* | *p* |
| Alert/Drowsy | 18.6 (21.3) | 25.6 (17.9) | 24.0 (17.7) | 32.4 (20.2) | 5.90 | *0.02 | 0.05 | >0.20 |
| Calm/Excited | 30.2 (24.2) | 34.4 (18.4) | 37.3 (23.1) | 40.6 (22.9) | 0.78 | >0.20 | 0.01 | >0.20 |
| Strong/Feeble | 25.0 (19.6) | 29.3 (18.3) | 29.7 (20.4) | 28.9 (17.5) | 0.38 | >0.20 | 0.85 | >0.20 |
| Fuzzy/Clear-headed | 77.0 (21.2) | 67.0 (31.5) | 75.6 (24.0) | 68.5 (27.4) | 4.77 | *0.03 | 0.14 | >0.20 |
| Well-coordinated/Clumsy | 25.3 (27.6) | 31.1 (26.4) | 26.2 (24.3) | 31.8 (28.0) | 1.66 | >0.20 | 0.00 | >0.20 |
| Lethargic/Energetic | 65.3 (18.3) | 58.5 (23.5) | 61.1 (17.8) | 49.6 (27.4) | 7.03 | *0.01 | 0.45 | >0.20 |
| Contented/Discontented | 25.2 (25.0) | 27.8 (24.6) | 18.7 (16.1) | 29.6 (22.4) | 4.88 | *0.03 | 1.79 | >0.20 |
| Troubled/Tranquil | 69.8 (23.0) | 68.8 (25.3) | 75.6 (20.9) | 60.1 (28.2) | 5.36 | *0.03 | 4.10 | 0.06 |
| Mentally-slow/Quick-witted | 76.1 (16.9) | 66.7 (22.9) | 67.0 (21.6) | 60.1 (24.4) | 7.89 | *0.01 | 0.20 | >0.20 |
| Tense/Relaxed | 71.0 (22.9) | 68.0 (23.3) | 71.5 (23.6) | 63.0 (24.4) | 3.99 | 0.05 | 0.89 | >0.20 |
| Attentive/Dreamy | 22.9 (16.3) | 32.1 (19.2) | 25.0 (20.0) | 36.5 (22.2) | 10.87 | *0.00 | 0.14 | >0.20 |
| Incompetent/Proficient | 76.6 (19.0) | 67.1 (22.3) | 73.4 (21.2) | 69.3 (19.2) | 6.51 | *0.01 | 1.00 | >0.20 |
| Happy/Sad | 24.3 (22.0) | 25.0 (18.2) | 19.2 (16.1) | 31.8 (23.7) | 3.68 | 0.06 | 2.92 | 0.09 |
| Antagonistic/Friendly | 78.2 (18.3) | 71.6 (27.4) | 82.4 (23.8) | 71.8 (29.4) | 4.58 | *0.04 | 0.25 | >0.20 |
| Interested/Bored | 18.8 (18.5) | 27.5 (23.7) | 23.0 (21.9) | 26.6 (19.5) | 4.27 | *0.04 | 0.74 | >0.20 |
| Withdrawn/Sociable | 74.6 (18.5) | 63.7 (24.5) | 71.6 (25.6) | 69.0 (25.3) | 3.07 | 0.09 | 1.18 | >0.20 |

^Values are means with (standard deviations). An asterisk (*) highlights a statistically significant difference between the simvastatin and placebo groups.^

##### S4 – Descriptive statistics for neuropsychological tasks

Outcomes measured are defined in the Methods. Values are means and standard errors of the mean, unless indicated otherwise.

***FERT***

| **FERT, accuracy** |  |  |  |  |  |  |  |
| --- | --- | --- | --- | --- | --- | --- | --- |
|  | **Placebo** |  | **Simvastatin** |  |  |  |  |
|  | N= 26 |  | N= 26 |  |  |  |  |
| Sad | 66.33 | 2.02 | 63.57 | 2.02 |  |  |  |
| Fear | 32.79 | 3.16 | 34.81 | 3.16 |  |  |  |
| Happy | 71.82 | 1.47 | 74.89 | 1.47 |  |  |  |
| Surprise | 69.72 | 1.47 | 72.56 | 1.47 |  |  |  |
| Disgust | 44.02 | 1.93 | 41.96 | 1.93 |  |  |  |
| Anger | 59.05 | 2.29 | 57.86 | 2.29 |  | *F* | *p* |
| Neutral | 83.85 | 2.62 | 87.09 | 2.62 |  | 0.79 | 0.56 |
|  |  |  |  |  |  |  |  |
| **FERT, reaction times** |  |  |  |  |  |  |  |
|  | **Placebo** |  | **Simvastatin** |  |  |  |  |
|  | N= 26 |  | N= 27 |  |  |  |  |
| Sad | 1.48 | 0.07 | 1.51 | 0.07 |  |  |  |
| Fear | 2.15 | 0.11 | 2.14 | 0.10 |  |  |  |
| Happy | 1.50 | 0.06 | 1.46 | 0.06 |  |  |  |
| Surprise | 1.48 | 0.06 | 1.44 | 0.06 |  |  |  |
| Disgust | 1.57 | 0.06 | 1.66 | 0.06 |  |  |  |
| Anger | 1.69 | 0.07 | 1.69 | 0.07 |  | *F* | *p* |
| Neutral | 1.22 | 0.07 | 1.24 | 0.07 |  | 0.51 | 0.74 |
|  |  |  |  |  |  |  |  |
| **FERT, misclassification** |  |  |  |  |  |  |  |
|  | **Placebo** |  | **Simvastatin** |  |  |  |  |
|  | N= 25 |  | N= 27 |  |  |  |  |
| Sad | 14.55 | 1.15 | 12.34 | 1.10 |  |  |  |
| Fear | 5.57 | 0.77 | 5.13 | 0.74 |  |  |  |
| Happy | 1.53 | 0.49 | 1.81 | 0.47 |  |  |  |
| Surprise | 12.13 | 0.98 | 12.06 | 0.94 |  |  |  |
| Disgust | 5.33 | 0.86 | 6.20 | 0.82 |  |  |  |
| Anger | 7.00 | 0.77 | 7.85 | 0.74 |  | *F* | *p* |
| Neutral | 53.89 | 2.04 | 54.60 | 1.97 |  | 0.43 | 0.71 |
|  |  |  |  |  |  |  |  |
| **FERT, accuracy pos/neg** |  |  |  |  |  |  |  |
|  | **Placebo** |  | **Simvastatin** |  |  |  |  |
|  | N= 26 |  | N= 27 |  |  |  |  |
| Positive | 70.77 | 1.30 | 72.66 | 1.28 |  | *F* | *p* |
| Negative | 50.55 | 1.47 | 49.33 | 1.45 |  | 1.40 | 0.24 |
|  |  |  |  |  |  |  |  |
| **FERT, d'** |  |  |  |  |  |  |  |
|  | **Placebo** |  | **Simvastatin** |  |  |  |  |
|  | N= 26 |  | N= 27 |  |  |  |  |
| Sad | 0.88 | 0.01 | 0.88 | 0.01 |  |  |  |
| Fear | 0.80 | 0.01 | 0.81 | 0.01 |  |  |  |
| Happy | 0.93 | 0.01 | 0.93 | 0.01 |  |  |  |
| Surprise | 0.90 | 0.00 | 0.91 | 0.00 |  |  |  |
| Disgust | 0.84 | 0.01 | 0.83 | 0.01 |  |  |  |
| Anger | 0.88 | 0.01 | 0.87 | 0.01 |  | *F* | *p* |
| Neutral | 0.88 | 0.01 | 0.89 | 0.01 |  | 0.75 | 0.55 |
|  |  |  |  |  |  |  |  |
| **FERT, beta** |  |  |  |  |  |  |  |
|  | **Placebo** |  | **Simvastatin** |  |  |  |  |
|  | N= 26 |  | N= 27 |  |  |  |  |
| Sad | 0.53 | 0.04 | 0.60 | 0.04 |  |  |  |
| Fear | 0.73 | 0.04 | 0.79 | 0.04 |  |  |  |
| Happy | 0.90 | 0.03 | 0.91 | 0.03 |  |  |  |
| Surprise | 0.58 | 0.03 | 0.57 | 0.03 |  |  |  |
| Disgust | 0.82 | 0.03 | 0.79 | 0.03 |  |  |  |
| Anger | 0.75 | 0.03 | 0.73 | 0.02 |  | *F* | *p* |
| Neutral | -0.30 | 0.09 | -0.39 | 0.09 |  | 0.74 | 0.50 |

***FERT, subgroup analyses***

***Gender***

Males N= 26, Females N= 27

| **FERT, gender, accuracy** |  |  |  |  |  |  |  |
| --- | --- | --- | --- | --- | --- | --- | --- |
|  |  | ***Placebo/Simvastatin*** |  |  |  |  |  |
|  |  | *MD* | *SE* | *p* |  |  |  |
| *Sad* | Male | 2.34 | 3.80 | 0.54 |  |  |  |
|  | Female | 3.18 | 3.80 | 0.41 |  |  |  |
| *Fear* | Male | 1.05 | 6.32 | 0.87 |  |  |  |
|  | Female | -5.11 | 6.32 | 0.42 |  |  |  |
| *Happy* | Male | -1.69 | 2.97 | 0.57 |  |  |  |
|  | Female | -4.45 | 2.97 | 0.14 |  |  |  |
| *Surprise* | Male | -3.11 | 2.99 | 0.30 |  |  |  |
|  | Female | -2.57 | 2.99 | 0.40 |  |  |  |
| *Disgust* | Male | 2.79 | 3.92 | 0.48 |  |  |  |
|  | Female | 1.35 | 3.92 | 0.73 |  |  |  |
| *Anger* | Male | 2.64 | 4.56 | 0.57 |  |  |  |
|  | Female | -0.26 | 4.56 | 0.96 |  |  |  |
| *Neutral* | Male | -1.11 | 5.32 | 0.84 |  | *F* | *p* |
|  | Female | -5.39 | 5.32 | 0.32 |  | 0.18 | 0.96 |
|  |  |  |  |  |  |  |  |
| **FERT, gender, reaction times** |  |  |  |  |  |  |  |
|  |  | ***Placebo/Simvastatin*** |  |  |  |  |  |
|  |  | *MD* | *SE* | *p* |  |  |  |
| *Sad* | Male | -0.08 | 0.13 | 0.53 |  |  |  |
|  | Female | 0.00 | 0.13 | 0.99 |  |  |  |
| *Fear* | Male | -0.09 | 0.19 | 0.63 |  |  |  |
|  | Female | 0.07 | 0.18 | 0.69 |  |  |  |
| *Happy* | Male | 0.04 | 0.11 | 0.73 |  |  |  |
|  | Female | 0.02 | 0.11 | 0.86 |  |  |  |
| *Surprise* | Male | 0.06 | 0.11 | 0.60 |  |  |  |
|  | Female | 0.01 | 0.11 | 0.93 |  |  |  |
| *Disgust* | Male | -0.11 | 0.11 | 0.30 |  |  |  |
|  | Female | -0.08 | 0.10 | 0.43 |  |  |  |
| *Anger* | Male | 0.03 | 0.13 | 0.85 |  |  |  |
|  | Female | -0.05 | 0.13 | 0.72 |  |  |  |
| *Neutral* | Male | -0.12 | 0.13 | 0.36 |  | *F* | *p* |
|  | Female | 0.07 | 0.12 | 0.58 |  | 0.73 | 0.58 |

| **FERT, gender, misclassifications** | | |  | | | | |  | | | | | | | | | |  | |  | | | | | | | |  | | | | | | | | |  |  | | |
| --- | --- | --- | --- | --- | --- | --- | --- | --- | --- | --- | --- | --- | --- | --- | --- | --- | --- | --- | --- | --- | --- | --- | --- | --- | --- | --- | --- | --- | --- | --- | --- | --- | --- | --- | --- | --- | --- | --- | --- | --- |
|  | | |  | | | | | ***Placebo/Simvastatin*** | | | | | | | | | |  | |  | | | | | | | |  | | | | | | | | |  |  | | |
|  | | |  | | | | | *MD* | | | | | | | | | | *SE* | | *p* | | | | | | | |  | | | | | | | | |  |  | | |
| Sad | | | Male | | | | | -0.86 | | | | | | | | | | 1.99 | | 0.67 | | | | | | | |  | | | | | | | | |  |  | | |
|  | | | Female | | | | | 5.04 | | | | | | | | | | 1.92 | | 0.01 | | | | | | | |  | | | | | | | | |  |  | | |
| Fear | | | Male | | | | | -0.42 | | | | | | | | | | 1.56 | | 0.79 | | | | | | | |  | | | | | | | | |  |  | | |
|  | | | Female | | | | | 1.23 | | | | | | | | | | 1.50 | | 0.42 | | | | | | | |  | | | | | | | | |  |  | | |
| Happy | | | Male | | | | | -0.46 | | | | | | | | | | 1.00 | | 0.65 | | | | | | | |  | | | | | | | | |  |  | | |
|  | | | Female | | | | | -0.12 | | | | | | | | | | 0.96 | | 0.90 | | | | | | | |  | | | | | | | | |  |  | | |
| Surprise | | | Male | | | | | -0.72 | | | | | | | | | | 1.99 | | 0.72 | | | | | | | |  | | | | | | | | |  |  | | |
|  | | | Female | | | | | 0.80 | | | | | | | | | | 1.92 | | 0.68 | | | | | | | |  | | | | | | | | |  |  | | |
| Disgust | | | Male | | | | | -1.49 | | | | | | | | | | 1.74 | | 0.40 | | | | | | | |  | | | | | | | | |  |  | | |
|  | | | Female | | | | | -0.29 | | | | | | | | | | 1.67 | | 0.86 | | | | | | | |  | | | | | | | | |  |  | | |
| Anger | | | Male | | | | | -0.93 | | | | | | | | | | 1.54 | | 0.55 | | | | | | | |  | |  |  | | | | | | | | | |
|  | | | Female | | | | | -0.77 | | | | | | | | | | 1.48 | | 0.60 | | | | | | | |  | |  |  | | | | | | | | | |
| Neutral | | | Male | | | | | 4.87 | | | | | | | | | | 3.99 | | 0.23 | | | | | | | |  | | *F* | *p* | | | | | | | | | |
|  | | | Female | | | | | -5.88 | | | | | | | | | | 3.84 | | 0.13 | | | | | | | |  | | 2.50 | 0.07 | | | | | | | | | |
|  | | |  | | | | |  | | | | | | | | | |  | |  | | | | | | | |  | | | | | | | | |  |  | | |
|  | *MD* | | | | | | *SE* | | | F | | | | | *p* | | *η^2^* | | | | | | |  |  |  |  |  |  |  |  |  |  |  |  |  |  |  |  |  |
| *Female, sad* | 5.04 | | | | | | 1.92 | | | 6.91 | | | | | 0.01 | | 0.07 | | | | | | |  |  |  |  |  |  |  |  |  |  |  |  |  |  |  |  |  |
|  | | | | | |  | | | | | |  | | | |  | | | | |  | | | | | |  | | | | |  |  | | | |  |  |  |  |
| **FERT, gender, accuracy positive/negative** | | | | | | | | |  | | | |  | | | |  | | | | |  | | | |  | | |  | | | | |  |  |  |  |  |  |  |
|  | | | |  | | | | | ***Placebo/Simvastatin*** | | | |  | | | |  | | | | |  | | | |  | | |  | | | | |  |  |  |  |  |  |  |
|  | | | |  | | | | | *MD* | | | | *SE* | | | | *p* | | | | |  | | | |  | | |  | | | | |  |  |  |  |  |  |  |
| *Positive* | | | | Male | | | | | -2.40 | | | | 2.60 | | | | 0.36 | | | | |  | | | |  | | |  | | | | |  |  |  |  |  |  |  |
|  | | | | Female | | | | | -1.50 | | | | 2.55 | | | | 0.56 | | | | |  | | | |  | | |  | | | | |  |  |  |  |  |  |  |
| *Negative* | | | | Male | | | | | 2.21 | | | | 2.83 | | | | 0.44 | | | | |  | | | | *F* | | | *p* | | | | |  |  |  |  |  |  |  |
|  | | | | Female | | | | | 0.45 | | | | 2.78 | | | | 0.87 | | | | |  | | | | 0.30 | | | 0.59 | | | | |  |  |  |  |  |  |  |
|  | | | |  | | | | |  | | | |  | | | |  | | | | |  | | | |  | | |  | | | | |  |  |  |  |  |  |  |
| **FERT, gender, d'** | | | |  | | | | |  | | | |  | | | |  | | | | |  | | | |  | | |  | | | | |  |  |  |  |  |  |  |
|  | | | |  | | | | | ***Placebo/Simvastatin*** | | | |  | | | |  | | | | |  | | | |  | | |  | | | | |  |  |  |  |  |  |  |
|  | | | |  | | | | | *MD* | | | | *SE* | | | | *p* | | | | |  | | | |  | | |  | | | | |  |  |  |  |  |  |  |
| *Sad* | | | | Male | | | | | 0.01 | | | | 0.01 | | | | 0.46 | | | | |  | | | |  | | |  | | | | |  |  |  |  |  |  |  |
|  | | | | Female | | | | | 0.00 | | | | 0.01 | | | | 0.88 | | | | |  | | | |  | | |  | | | | |  |  |  |  |  |  |  |
| *Fear* | | | | Male | | | | | 0.01 | | | | 0.03 | | | | 0.82 | | | | |  | | | |  | | |  | | | | |  |  |  |  |  |  |  |
|  | | | | Female | | | | | -0.03 | | | | 0.03 | | | | 0.35 | | | | |  | | | |  | | |  | | | | |  |  |  |  |  |  |  |
| *Happy* | | | | Male | | | | | -0.01 | | | | 0.01 | | | | 0.57 | | | | |  | | | |  | | |  | | | | |  |  |  |  |  |  |  |
|  | | | | Female | | | | | 0.00 | | | | 0.01 | | | | 0.72 | | | | |  | | | |  | | |  | | | | |  |  |  |  |  |  |  |
| *Surprise* | | | | Male | | | | | -0.01 | | | | 0.01 | | | | 0.60 | | | | |  | | | |  | | |  | | | | |  |  |  |  |  |  |  |
|  | | | | Female | | | | | -0.01 | | | | 0.01 | | | | 0.41 | | | | |  | | | |  | | |  | | | | |  |  |  |  |  |  |  |
| *Disgust* | | | | Male | | | | | 0.01 | | | | 0.02 | | | | 0.46 | | | | |  | | | |  | | |  | | | | |  |  |  |  |  |  |  |
|  | | | | Female | | | | | 0.01 | | | | 0.01 | | | | 0.55 | | | | |  | | | |  | | |  | | | | |  |  |  |  |  |  |  |
| *Anger* | | | | Male | | | | | 0.01 | | | | 0.01 | | | | 0.44 | | | | |  | | | |  | | |  | | | | |  |  |  |  |  |  |  |
|  | | | | Female | | | | | 0.01 | | | | 0.01 | | | | 0.65 | | | | |  | | | |  | | |  | | | | |  |  |  |  |  |  |  |
| *Neutral* | | | | Male | | | | | -0.01 | | | | 0.02 | | | | 0.70 | | | | |  | | | | *F* | | | *p* | | | | |  |  |  |  |  |  |  |
|  | | | | Female | | | | | -0.02 | | | | 0.02 | | | | 0.41 | | | | |  | | | | 0.31 | | | 0.85 | | | | |  |  |  |  |  |  |  |
|  | | | |  | | | | |  | | | |  | | | |  | | | | |  | | | |  | | |  | | | | |  |  |  |  |  |  |  |
| **FERT, gender, beta** | | | |  | | | | |  | | | |  | | | |  | | | | |  | | | |  | | |  | | | | |  |  |  |  |  |  |  |
|  | | | |  | | | | | ***Placebo/Simvastatin*** | | | |  | | | |  | | | | |  | | | |  | | |  | | | | |  |  |  |  |  |  |  |
|  | | | |  | | | | | *MD* | | | | *SE* | | | | *p* | | | | |  | | | |  | | |  | | | | |  |  |  |  |  |  |  |
| *Sad* | | | | Male | | | | | 0.01 | | | | 0.06 | | | | 0.85 | | | | |  | | | |  | | |  | | | | |  |  |  |  |  |  |  |
|  | | | | Female | | | | | -0.17 | | | | 0.06 | | | | 0.01 | | | | |  | | | |  | | |  | | | | |  |  |  |  |  |  |  |
| *Fear* | | | | Male | | | | | -0.02 | | | | 0.08 | | | | 0.79 | | | | |  | | | |  | | |  | | | | |  |  |  |  |  |  |  |
|  | | | | Female | | | | | -0.08 | | | | 0.08 | | | | 0.30 | | | | |  | | | |  | | |  | | | | |  |  |  |  |  |  |  |
| *Happy* | | | | Male | | | | | -0.02 | | | | 0.06 | | | | 0.66 | | | | |  | | | |  | | |  | | | | |  |  |  |  |  |  |  |
|  | | | | Female | | | | | 0.00 | | | | 0.06 | | | | 0.95 | | | | |  | | | |  | | |  | | | | |  |  |  |  |  |  |  |
| *Surprise* | | | | Male | | | | | 0.05 | | | | 0.07 | | | | 0.49 | | | | |  | | | |  | | |  | | | | |  |  |  |  |  |  |  |
|  | | | | Female | | | | | -0.01 | | | | 0.07 | | | | 0.91 | | | | |  | | | |  | | |  | | | | |  |  |  |  |  |  |  |
| *Disgust* | | | | Male | | | | | 0.05 | | | | 0.06 | | | | 0.38 | | | | |  | | | |  | | |  | | | | |  |  |  |  |  |  |  |
|  | | | | Female | | | | | 0.01 | | | | 0.06 | | | | 0.87 | | | | |  | | | |  | | |  | | | | |  |  |  |  |  |  |  |
| *Anger* | | | | Male | | | | | 0.01 | | | | 0.05 | | | | 0.79 | | | | |  | | | |  | | |  | | | | |  |  |  |  |  |  |  |
|  | | | | Female | | | | | 0.03 | | | | 0.05 | | | | 0.58 | | | | |  | | | |  | | |  | | | | |  |  |  |  |  |  |  |
| *Neutral* | | | | Male | | | | | -0.03 | | | | 0.17 | | | | 0.87 | | | | |  | | | | *F* | | | *p* | | | | |  |  |  |  |  |  |  |
|  | | | | Female | | | | | 0.21 | | | | 0.17 | | | | 0.23 | | | | |  | | | | 1.01 | | | 0.37 | | | | |  |  |  |  |  |  |  |
| **Subgroup analysis for females**  **FERT, female, accuracy** | | | | | |  | | | | | |  | | | |  | | | | |  | | | | | |  | | | | |  |  | | | |  |  |  |  |
|  | | | | | | **Placebo** | | | | | |  | | | | **Simvastatin** | | | | |  | | | | | |  | | | | |  |  | | | |  |  |  |  |
|  | | | | | | N= 13 | | | | | |  | | | | N= 13 | | | | |  | | | | | |  | | | | |  |  | | | |  |  |  |  |
| Sad | | | | | | 70.51 | | | | | | 2.57 | | | | 67.32 | | | | | 2.57 | | | | | |  | | | | |  |  | | | |  |  |  |  |
| Fear | | | | | | 34.03 | | | | | | 4.17 | | | | 39.14 | | | | | 4.17 | | | | | |  | | | | |  |  | | | |  |  |  |  |
| Happy | | | | | | 70.17 | | | | | | 2.09 | | | | 74.62 | | | | | 2.09 | | | | | |  | | | | |  |  | | | |  |  |  |  |
| Surprise | | | | | | 69.19 | | | | | | 1.85 | | | | 71.76 | | | | | 1.85 | | | | | |  | | | | |  |  | | | |  |  |  |  |
| Disgust | | | | | | 44.81 | | | | | | 2.52 | | | | 43.46 | | | | | 2.52 | | | | | |  | | | | |  |  | | | |  |  |  |  |
| Anger | | | | | | 60.75 | | | | | | 2.40 | | | | 61.01 | | | | | 2.40 | | | | | |  | | | | | *F* | *p* | | | |  |  |  |  |
| Neutral | | | | | | 82.31 | | | | | | 3.23 | | | | 87.69 | | | | | 3.23 | | | | | |  | | | | | 0.82 | 0.54 | | | |  |  |  |  |
|  | | | | | |  | | | | | |  | | | |  | | | | |  | | | | | |  | | | | |  |  | | | |  |  |  |  |
| **FERT, female, reaction times** | | | | | |  | | | | | |  | | | |  | | | | |  | | | | | |  | | | | |  |  | | | |  |  |  |  |
|  | | | | | | **Placebo** | | | | | |  | | | | **Simvastatin** | | | | |  | | | | | |  | | | | |  |  | | | |  |  |  |  |
|  | | | | | | N= 13 | | | | | |  | | | | N= 14 | | | | |  | | | | | |  | | | | |  |  | | | |  |  |  |  |
| Sad | | | | | | 1.38 | | | | | | 0.07 | | | | 1.38 | | | | | 0.07 | | | | | |  | | | | |  |  | | | |  |  |  |  |
| Fear | | | | | | 1.93 | | | | | | 0.07 | | | | 1.85 | | | | | 0.07 | | | | | |  | | | | |  |  | | | |  |  |  |  |
| Happy | | | | | | 1.39 | | | | | | 0.07 | | | | 1.37 | | | | | 0.07 | | | | | |  | | | | |  |  | | | |  |  |  |  |
| Surprise | | | | | | 1.39 | | | | | | 0.07 | | | | 1.38 | | | | | 0.06 | | | | | |  | | | | |  |  | | | |  |  |  |  |
| Disgust | | | | | | 1.44 | | | | | | 0.07 | | | | 1.52 | | | | | 0.06 | | | | | |  | | | | |  |  | | | |  |  |  |  |
| Anger | | | | | | 1.53 | | | | | | 0.07 | | | | 1.57 | | | | | 0.07 | | | | | |  | | | | | *F* | *p* | | | |  |  |  |  |
| Neutral | | | | | | 1.14 | | | | | | 0.06 | | | | 1.07 | | | | | 0.05 | | | | | |  | | | | | 0.92 | 0.48 | | | |  |  |  |  |
|  | | | | | |  | | | | | |  | | | |  | | | | |  | | | | | |  | | | | |  |  | | | |  |  |  |  |
| **FERT, female, misclassifications** | | | | | |  | | | | | |  | | | |  | | | | |  | | | | | |  | | | | |  |  | | | |  |  |  |  |
|  | | | | | | **Placebo** | | | | | |  | | | | **Simvastatin** | | | | |  | | | | | |  | | | | |  |  | | | |  |  |  |  |
|  | | | | | | N= 13 | | | | | |  | | | | N= 14 | | | | |  | | | | | |  | | | | |  |  | | | |  |  |  |  |
| Sad | | | | | | 18.49 | | | | | | 1.41 | | | | 13.45 | | | | | 1.36 | | | | | |  | | | | |  |  | | | |  |  |  |  |
| Fear | | | | | | 6.12 | | | | | | 0.81 | | | | 4.89 | | | | | 0.78 | | | | | |  | | | | |  |  | | | |  |  |  |  |
| Happy | | | | | | 1.36 | | | | | | 0.50 | | | | 1.48 | | | | | 0.48 | | | | | |  | | | | |  |  | | | |  |  |  |  |
| Surprise | | | | | | 12.28 | | | | | | 1.40 | | | | 11.48 | | | | | 1.35 | | | | | |  | | | | |  |  | | | |  |  |  |  |
| Disgust | | | | | | 5.36 | | | | | | 1.18 | | | | 5.65 | | | | | 1.14 | | | | | |  | | | | |  |  | | | |  |  |  |  |
| Anger | | | | | | 6.39 | | | | | | 1.03 | | | | 7.17 | | | | | 0.99 | | | | | |  | | | | | *F* | *p* | | | |  |  |  |  |
| Neutral | | | | | | 50.00 | | | | | | 2.32 | | | | 55.88 | | | | | 2.24 | | | | | |  | | | | | 2.57 | 0.05 | | | |  |  |  |  |
|  |  | | | | | |  | | |  | | | | |  | |  | | | | | | |  |  | | | | | | | | | |  |  | | |  |  |
|  | *MD* | | | | | | *SE* | | | F | | | | | *p* | | *η^2^* | | | | | | |  |  |  |  |  |  |  |  |  |  |  |  |  |  |  |  |  |
| *Sad* | 5.04 | | | | | | 1.96 | | | 6.60 | | | | | 0.02 | | 0.21 | | | | | | |  |  |  |  |  |  |  |  |  |  |  |  |  |  |  |  |  |
|  | |  |  |  |  |  |  |  |  |  |  |  |  |  |  |  |  |  |  |  |  |  |  |  |  |  |  |  |  |  |  |  |  |  |  |  |  |  |  |  |
| ***Positive as sad*** | | 1.50 | | | 0.62 | | | | | | 5.79 | | | 0.02 | | | | | 0.19 | | | |  |  |  |  |  |  |  |  |  |  |  |  |  |  |  |  |  |  |
|  |  | | | | | |  | | |  | | | | |  | |  | | | | | | |  |  |  |  |  |  |  |  |  |  |  |  |  |  |  |  |  |
| **FERT, female, accuracy pos/neg** | | | | | | | | | | | |  | | | |  | | | | |  | | | | | |  | | | | |  |  | | | | | | | |
|  | | | | | | **Placebo** | | | | | |  | | | | **Simvastatin** | | | | |  | | | | | |  | | | | |  |  | | | | | | | |
|  | | | | | | N= 13 | | | | | |  | | | | N= 14 | | | | |  | | | | | |  | | | | |  |  | | | | | | | |
| Positive | | | | | | 69.68 | | | | | | 2.00 | | | | 71.18 | | | | | 1.93 | | | | | |  | | | | | *F* | *p* | | | | | | | |
| Negative | | | | | | 52.52 | | | | | | 1.74 | | | | 52.08 | | | | | 1.67 | | | | | |  | | | | | 0.46 | 0.50 | | | | | | | |
|  | | | | | |  | | | | | |  | | | |  | | | | |  | | | | | |  | | | | |  |  | | | | | | | |
| **FERT, female, d'** | | | | | |  | | | | | |  | | | |  | | | | |  | | | | | |  | | | | |  |  | | | | | | | |
|  | | | | | | **Placebo** | | | | | |  | | | | **Simvastatin** | | | | |  | | | | | |  | | | | |  |  | | | | | | | |
|  | | | | | | N= 13 | | | | | |  | | | | N= 14 | | | | |  | | | | | |  | | | | |  |  | | | | | | | |
| Sad | | | | | | 0.89 | | | | | | 0.01 | | | | 0.89 | | | | | 0.01 | | | | | |  | | | | |  |  | | | | | | | |
| Fear | | | | | | 0.80 | | | | | | 0.02 | | | | 0.83 | | | | | 0.02 | | | | | |  | | | | |  |  | | | | | | | |
| Happy | | | | | | 0.92 | | | | | | 0.01 | | | | 0.93 | | | | | 0.01 | | | | | |  | | | | |  |  | | | | | | | |
| Surprise | | | | | | 0.90 | | | | | | 0.01 | | | | 0.90 | | | | | 0.01 | | | | | |  | | | | |  |  | | | | | | | |
| Disgust | | | | | | 0.84 | | | | | | 0.01 | | | | 0.83 | | | | | 0.01 | | | | | |  | | | | |  |  | | | | | | | |
| Anger | | | | | | 0.89 | | | | | | 0.01 | | | | 0.88 | | | | | 0.01 | | | | | |  | | | | | *F* | *p* | | | | | | | |
| Neutral | | | | | | 0.88 | | | | | | 0.01 | | | | 0.89 | | | | | 0.01 | | | | | |  | | | | | 0.93 | 0.46 | | | | | | | |
|  | | | | | |  | | | | | |  | | | |  | | | | |  | | | | | |  | | | | |  |  | | | | | | | |
| **FERT, female, beta** | | | | | |  | | | | | |  | | | |  | | | | |  | | | | | |  | | | | |  |  | | | | | | | |
|  | | | | | | **Placebo** | | | | | |  | | | | **Simvastatin** | | | | |  | | | | | |  | | | | |  |  | | | | | | | |
|  | | | | | | N= 13 | | | | | |  | | | | N= 14 | | | | |  | | | | | |  | | | | |  |  | | | | | | | |
| Sad | | | | | | 0.40 | | | | | | 0.05 | | | | 0.57 | | | | | 0.05 | | | | | |  | | | | |  |  | | | | | | | |
| Fear | | | | | | 0.74 | | | | | | 0.04 | | | | 0.82 | | | | | 0.04 | | | | | |  | | | | |  |  | | | | | | | |
| Happy | | | | | | 0.94 | | | | | | 0.02 | | | | 0.93 | | | | | 0.02 | | | | | |  | | | | |  |  | | | | | | | |
| Surprise | | | | | | 0.59 | | | | | | 0.05 | | | | 0.60 | | | | | 0.04 | | | | | |  | | | | |  |  | | | | | | | |
| Disgust | | | | | | 0.82 | | | | | | 0.04 | | | | 0.81 | | | | | 0.04 | | | | | |  | | | | |  |  | | | | | | | |
| Anger | | | | | | 0.79 | | | | | | 0.03 | | | | 0.76 | | | | | 0.03 | | | | | |  | | | | | *F* | *p* | | | | | | | |
| Neutral | | | | | | -0.17 | | | | | | 0.10 | | | | -0.38 | | | | | 0.10 | | | | | |  | | | | | 2.42 | 0.08 | | | | | | | |

***BMI***

Normoweight N= 44, Overweight N= 9

| **FERT, BMI, accuracy** | |  |  |  |  |  |  |
| --- | --- | --- | --- | --- | --- | --- | --- |
|  |  | ***Placebo/Simvastatin*** |  |  |  |  |  |
|  |  | *MD* | *SE* | *p* |  |  |  |
| *Sad* | Normoweight | 1.27 | 3.11 | 0.69 |  |  |  |
|  | Overweight | 8.74 | 6.84 | 0.21 |  |  |  |
| *Fear* | Normoweight | -2.50 | 4.95 | 0.62 |  |  |  |
|  | Overweight | -1.27 | 10.89 | 0.91 |  |  |  |
| *Happy* | Normoweight | -3.13 | 2.31 | 0.18 |  |  |  |
|  | Overweight | -2.10 | 5.08 | 0.68 |  |  |  |
| *Surprise* | Normoweight | -2.46 | 2.32 | 0.30 |  |  |  |
|  | Overweight | -4.17 | 5.10 | 0.42 |  |  |  |
| *Disgust* | Normoweight | 1.47 | 3.05 | 0.63 |  |  |  |
|  | Overweight | 4.45 | 6.71 | 0.51 |  |  |  |
| *Anger* | Normoweight | 2.40 | 3.34 | 0.48 |  |  |  |
|  | Overweight | -7.10 | 7.35 | 0.34 |  |  |  |
| *Neutral* | Normoweight | -3.68 | 4.10 | 0.37 |  | *F* | *p* |
|  | Overweight | 0.00 | 9.01 | 1.00 |  | 0.46 | 0.78 |
|  |  |  |  |  |  |  |  |
| **FERT, BMI, reaction times** | |  |  |  |  |  |  |
|  |  | ***Placebo/Simvastatin*** |  |  |  |  |  |
|  |  | *MD* | *SE* | *p* |  |  |  |
| *Sad* | Normoweight | -0.03 | 0.11 | 0.77 |  |  |  |
|  | Overweight | -0.05 | 0.24 | 0.82 |  |  |  |
| *Fear* | Normoweight | 0.02 | 0.17 | 0.91 |  |  |  |
|  | Overweight | -0.04 | 0.37 | 0.90 |  |  |  |
| *Happy* | Normoweight | 0.05 | 0.09 | 0.63 |  |  |  |
|  | Overweight | -0.02 | 0.21 | 0.94 |  |  |  |
| *Surprise* | Normoweight | 0.03 | 0.09 | 0.75 |  |  |  |
|  | Overweight | 0.06 | 0.20 | 0.77 |  |  |  |
| *Disgust* | Normoweight | -0.07 | 0.09 | 0.46 |  |  |  |
|  | Overweight | -0.19 | 0.20 | 0.36 |  |  |  |
| *Anger* | Normoweight | 0.03 | 0.11 | 0.81 |  |  |  |
|  | Overweight | -0.14 | 0.24 | 0.56 |  |  |  |
| *Neutral* | Normoweight | -0.01 | 0.11 | 0.94 |  | *F* | *p* |
|  | Overweight | -0.07 | 0.24 | 0.78 |  | 0.15 | 0.96 |
|  |  |  |  |  |  |  |  |
| **FERT, BMI, misclassifications** | | |  |  |  |  |  |
|  |  | ***Placebo/Simvastatin*** |  |  |  |  |  |
|  |  | *MD* | *SE* | *p* |  |  |  |
| *Sad* | Normoweight | 2.50 | 1.78 | 0.17 |  |  |  |
|  | Overweight | 0.68 | 3.91 | 0.86 |  |  |  |
| *Fear* | Normoweight | 0.83 | 1.19 | 0.49 |  |  |  |
|  | Overweight | -1.45 | 2.61 | 0.58 |  |  |  |
| *Happy* | Normoweight | -0.61 | 0.74 | 0.41 |  |  |  |
|  | Overweight | 1.49 | 1.63 | 0.37 |  |  |  |
| *Surprise* | Normoweight | 0.68 | 1.51 | 0.65 |  |  |  |
|  | Overweight | -2.76 | 3.32 | 0.41 |  |  |  |
| *Disgust* | Normoweight | -2.38 | 1.22 | 0.06 |  |  |  |
|  | Overweight | 6.51 | 2.69 | 0.02 |  |  |  |
| *Anger* | Normoweight | -0.78 | 1.17 | 0.51 |  |  |  |
|  | Overweight | -1.48 | 2.57 | 0.57 |  |  |  |
| *Neutral* | Normoweight | -0.24 | 3.18 | 0.94 |  | *F* | *p* |
|  | Overweight | -2.99 | 6.99 | 0.67 |  | 0.94 | 0.41 |
|  |  |  |  |  |  |  |  |
| **FERT,BMI, accuracy positive/negative** | | |  |  |  |  |  |
|  |  | ***Placebo/Simvastatin*** |  |  |  |  |  |
|  |  | *MD* | *SE* | *p* |  |  |  |
| *Positive* | Normoweight | -1.51 | 2.00 | 0.45 |  |  |  |
|  | Overweight | -3.14 | 4.45 | 0.48 |  |  |  |
| *Negative* | Normoweight | 0.99 | 2.19 | 0.65 |  | *F* | *p* |
|  | Overweight | 1.20 | 4.87 | 0.81 |  | 0.08 | 0.78 |
|  |  |  |  |  |  |  |  |
| **FERT, BMI, d'** | |  |  |  |  |  |  |
|  |  | ***Placebo/Simvastatin*** |  |  |  |  |  |
|  |  | *MD* | *SE* | *p* |  |  |  |
| *Sad* | Normoweight | 0.00 | 0.01 | 0.84 |  |  |  |
|  | Overweight | 0.03 | 0.02 | 0.22 |  |  |  |
| *Fear* | Normoweight | -0.01 | 0.02 | 0.54 |  |  |  |
|  | Overweight | 0.00 | 0.05 | 0.95 |  |  |  |
| *Happy* | Normoweight | 0.00 | 0.01 | 0.65 |  |  |  |
|  | Overweight | -0.01 | 0.02 | 0.66 |  |  |  |
| *Surprise* | Normoweight | -0.01 | 0.01 | 0.41 |  |  |  |
|  | Overweight | -0.01 | 0.02 | 0.69 |  |  |  |
| *Disgust* | Normoweight | 0.01 | 0.01 | 0.20 |  |  |  |
|  | Overweight | -0.02 | 0.03 | 0.53 |  |  |  |
| *Anger* | Normoweight | 0.01 | 0.01 | 0.25 |  |  |  |
|  | Overweight | -0.02 | 0.02 | 0.47 |  |  |  |
| *Neutral* | Normoweight | -0.01 | 0.02 | 0.35 |  | *F* | *p* |
|  | Overweight | 0.00 | 0.03 | 0.95 |  | 0.67 | 0.60 |
|  |  |  |  |  |  |  |  |
| **FERT, BMI, beta** | |  |  |  |  |  |  |
|  |  | ***Placebo/Simvastatin*** |  |  |  |  |  |
|  |  | *MD* | *SE* | *p* |  |  |  |
| *Sad* | Normoweight | -0.08 | 0.06 | 0.13 |  |  |  |
|  | Overweight | -0.04 | 0.12 | 0.77 |  |  |  |
| *Fear* | Normoweight | -0.06 | 0.06 | 0.30 |  |  |  |
|  | Overweight | -0.01 | 0.13 | 0.92 |  |  |  |
| *Happy* | Normoweight | 0.01 | 0.04 | 0.90 |  |  |  |
|  | Overweight | -0.11 | 0.09 | 0.27 |  |  |  |
| *Surprise* | Normoweight | 0.00 | 0.05 | 0.95 |  |  |  |
|  | Overweight | 0.12 | 0.12 | 0.30 |  |  |  |
| *Disgust* | Normoweight | 0.08 | 0.04 | 0.05 |  |  |  |
|  | Overweight | -0.22 | 0.09 | 0.01 |  |  |  |
| *Anger* | Normoweight | 0.02 | 0.04 | 0.68 |  |  |  |
|  | Overweight | 0.05 | 0.09 | 0.61 |  |  |  |
| *Neutral* | Normoweight | 0.09 | 0.14 | 0.53 |  | *F* | *p* |
|  | Overweight | 0.08 | 0.30 | 0.79 |  | 0.67 | 0.52 |

***hs-CRP (research visit)***

hs-CRP<1 N= 38, hs-CRP≥1 N= 15

| **FERT, hs-CRP, accuracy** | |  |  |  |  |  |  |
| --- | --- | --- | --- | --- | --- | --- | --- |
|  |  | ***Placebo/Simvastatin*** |  |  |  |  |  |
|  |  | *MD* | *SE* | *p* |  |  |  |
| *Sad* | hs-CRP<1 | 5.39 | 3.35 | 0.11 |  |  |  |
|  | hs-CRP≥1 | -0.84 | 5.48 | 0.88 |  |  |  |
| *Fear* | hs-CRP<1 | -1.28 | 5.42 | 0.82 |  |  |  |
|  | hs-CRP≥1 | -1.24 | 8.87 | 0.89 |  |  |  |
| *Happy* | hs-CRP<1 | -2.57 | 2.53 | 0.32 |  |  |  |
|  | hs-CRP≥1 | -3.66 | 4.14 | 0.38 |  |  |  |
| *Surprise* | hs-CRP<1 | -4.33 | 2.49 | 0.09 |  |  |  |
|  | hs-CRP≥1 | -0.70 | 4.07 | 0.86 |  |  |  |
| *Disgust* | hs-CRP<1 | 2.43 | 3.31 | 0.47 |  |  |  |
|  | hs-CRP≥1 | 1.21 | 5.42 | 0.83 |  |  |  |
| *Anger* | hs-CRP<1 | -1.24 | 3.88 | 0.75 |  |  |  |
|  | hs-CRP≥1 | 9.11 | 6.34 | 0.16 |  |  |  |
| *Neutral* | hs-CRP<1 | -4.11 | 4.43 | 0.36 |  | *F* | *p* |
|  | hs-CRP≥1 | -2.08 | 7.25 | 0.78 |  | 0.56 | 0.71 |
|  |  |  |  |  |  |  |  |
| **FERT, hs-CRP, reaction times** | | |  |  |  |  |  |
|  |  | ***Placebo/Simvastatin*** |  |  |  |  |  |
|  |  | *MD* | *SE* | *p* |  |  |  |
| *Sad* | hs-CRP<1 | 0.01 | 0.11 | 0.94 |  |  |  |
|  | hs-CRP≥1 | -0.11 | 0.19 | 0.58 |  |  |  |
| *Fear* | hs-CRP<1 | -0.03 | 0.18 | 0.88 |  |  |  |
|  | hs-CRP≥1 | 0.00 | 0.29 | 0.99 |  |  |  |
| *Happy* | hs-CRP<1 | 0.06 | 0.10 | 0.58 |  |  |  |
|  | hs-CRP≥1 | -0.01 | 0.17 | 0.94 |  |  |  |
| *Surprise* | hs-CRP<1 | 0.10 | 0.10 | 0.32 |  |  |  |
|  | hs-CRP≥1 | -0.03 | 0.16 | 0.84 |  |  |  |
| *Disgust* | hs-CRP<1 | 0.00 | 0.10 | 1.00 |  |  |  |
|  | hs-CRP≥1 | -0.28 | 0.16 | 0.09 |  |  |  |
| *Anger* | hs-CRP<1 | 0.09 | 0.12 | 0.45 |  |  |  |
|  | hs-CRP≥1 | -0.25 | 0.20 | 0.21 |  |  |  |
| *Neutral* | hs-CRP<1 | 0.03 | 0.11 | 0.82 |  | *F* | *p* |
|  | hs-CRP≥1 | -0.04 | 0.19 | 0.84 |  | 0.86 | 0.49 |
|  |  |  |  |  |  |  |  |
| **FERT, hs-CRP, misclassifications** | | |  |  |  |  |  |
|  |  | ***Placebo/Simvastatin*** |  |  |  |  |  |
|  |  | *MD* | *SE* | *p* |  |  |  |
| *Sad* | hs-CRP<1 | 5.05 | 1.79 | 0.01 |  |  |  |
|  | hs-CRP≥1 | -3.48 | 2.92 | 0.24 |  |  |  |
| *Fear* | hs-CRP<1 | 0.93 | 1.56 | 0.79 |  |  |  |
|  | hs-CRP≥1 | 1.66 | 2.12 | 0.44 |  |  |  |
| *Happy* | hs-CRP<1 | -0.02 | 0.84 | 0.98 |  |  |  |
|  | hs-CRP≥1 | -0.80 | 1.36 | 0.56 |  |  |  |
| *Surprise* | hs-CRP<1 | -0.34 | 1.66 | 0.84 |  |  |  |
|  | hs-CRP≥1 | 0.57 | 2.71 | 0.84 |  |  |  |
| *Disgust* | hs-CRP<1 | -2.29 | 1.40 | 0.11 |  |  |  |
|  | hs-CRP≥1 | 3.03 | 2.28 | 0.19 |  |  |  |
| *Anger* | hs-CRP<1 | -1.89 | 1.23 | 0.13 |  |  |  |
|  | hs-CRP≥1 | 3.03 | 1.99 | 0.14 |  |  |  |
| *Neutral* | hs-CRP<1 | -0.64 | 3.40 | 0.85 |  | *F* | *p* |
|  | hs-CRP≥1 | -4.02 | 5.54 | 0.47 |  | 1.64 | 0.19 |
|  |  |  |  |  |  |  |  |
| **FERT, hs-CRP, accuracy positive/negative** | | | |  |  |  |  |
|  |  | ***Placebo/Simvastatin*** |  |  |  |  |  |
|  |  | *MD* | *SE* | *p* |  |  |  |
| *Positive* | hs-CRP<1 | -2.40 | 2.60 | 0.36 |  |  |  |
|  | hs-CRP≥1 | -1.50 | 2.55 | 0.56 |  |  |  |
| *Negative* | hs-CRP<1 | 2.21 | 2.83 | 0.44 |  | *F* | *p* |
|  | hs-CRP≥1 | 0.45 | 2.78 | 0.87 |  | 0.46 | 0.64 |
|  |  |  |  |  |  |  |  |
| **FERT, hs-CRP, d'** | |  |  |  |  |  |  |
|  |  | ***Placebo/Simvastatin*** |  |  |  |  |  |
|  |  | *MD* | *SE* | *p* |  |  |  |
| *Sad* | hs-CRP<1 | 0.01 | 0.01 | 0.65 |  |  |  |
|  | hs-CRP≥1 | 0.01 | 0.02 | 0.72 |  |  |  |
| *Fear* | hs-CRP<1 | -0.01 | 0.02 | 0.78 |  |  |  |
|  | hs-CRP≥1 | -0.02 | 0.04 | 0.67 |  |  |  |
| *Happy* | hs-CRP<1 | 0.00 | 0.01 | 0.69 |  |  |  |
|  | hs-CRP≥1 | -0.01 | 0.01 | 0.60 |  |  |  |
| *Surprise* | hs-CRP<1 | -0.01 | 0.01 | 0.25 |  |  |  |
|  | hs-CRP≥1 | 0.00 | 0.01 | 0.84 |  |  |  |
| *Disgust* | hs-CRP<1 | 0.02 | 0.01 | 0.16 |  |  |  |
|  | hs-CRP≥1 | -0.01 | 0.02 | 0.59 |  |  |  |
| *Anger* | hs-CRP<1 | 0.00 | 0.01 | 0.71 |  |  |  |
|  | hs-CRP≥1 | 0.02 | 0.02 | 0.33 |  |  |  |
| *Neutral* | hs-CRP<1 | -0.01 | 0.02 | 0.37 |  | *F* | *p* |
|  | hs-CRP≥1 | 0.00 | 0.03 | 0.94 |  | 0.41 | 0.77 |
|  |  |  |  |  |  |  |  |
| **FERT, hs-CRP, beta** | |  |  |  |  |  |  |
|  |  | ***Placebo/Simvastatin*** |  |  |  |  |  |
|  |  | *MD* | *SE* | *p* |  |  |  |
| *Sad* | hs-CRP<1 | -0.16 | 0.06 | 0.01 |  |  |  |
|  | hs-CRP≥1 | 0.10 | 0.09 | 0.26 |  |  |  |
| *Fear* | hs-CRP<1 | -0.05 | 0.06 | 0.44 |  |  |  |
|  | hs-CRP≥1 | -0.07 | 0.11 | 0.49 |  |  |  |
| *Happy* | hs-CRP<1 | -0.04 | 0.05 | 0.43 |  |  |  |
|  | hs-CRP≥1 | 0.04 | 0.08 | 0.60 |  |  |  |
| *Surprise* | hs-CRP<1 | 0.04 | 0.06 | 0.50 |  |  |  |
|  | hs-CRP≥1 | 0.01 | 0.09 | 0.91 |  |  |  |
| *Disgust* | hs-CRP<1 | 0.08 | 0.05 | 0.09 |  |  |  |
|  | hs-CRP≥1 | -0.10 | 0.08 | 0.18 |  |  |  |
| *Anger* | hs-CRP<1 | 0.06 | 0.04 | 0.11 |  |  |  |
|  | hs-CRP≥1 | -0.13 | 0.07 | 0.06 |  |  |  |
| *Neutral* | hs-CRP<1 | 0.15 | 0.14 | 0.29 |  | *F* | *p* |
|  | hs-CRP≥1 | -0.03 | 0.24 | 0.89 |  | 1.35 | 0.27 |

***Family history***

NoFamHx N= 32, PosFamHx N= 20

| **FERT, famhx, accuracy** | |  |  |  |  |  |  |
| --- | --- | --- | --- | --- | --- | --- | --- |
|  |  | ***Placebo/Simvastatin*** |  |  |  |  |  |
|  |  | *MD* | *SE* | *p* |  |  |  |
| *Sad* | NoFamHx | 0.79 | 3.62 | 0.83 |  |  |  |
|  | PosFamHx | 5.03 | 4.59 | 0.28 |  |  |  |
| *Fear* | NoFamHx | 0.61 | 5.60 | 0.91 |  |  |  |
|  | PosFamHx | -7.87 | 7.11 | 0.27 |  |  |  |
| *Happy* | NoFamHx | 0.16 | 2.61 | 0.95 |  |  |  |
|  | PosFamHx | -8.17 | 3.31 | 0.02 |  |  |  |
| *Surprise* | NoFamHx | -2.17 | 2.70 | 0.43 |  |  |  |
|  | PosFamHx | -3.65 | 3.43 | 0.29 |  |  |  |
| *Disgust* | NoFamHx | -0.34 | 3.51 | 0.92 |  |  |  |
|  | PosFamHx | 5.63 | 4.46 | 0.21 |  |  |  |
| *Anger* | NoFamHx | 1.87 | 4.19 | 0.66 |  |  |  |
|  | PosFamHx | -0.41 | 5.32 | 0.94 |  |  |  |
| *Neutral* | NoFamHx | -0.90 | 4.69 | 0.85 |  | *F* | *p* |
|  | PosFamHx | -5.85 | 5.94 | 0.33 |  | 0.87 | 0.49 |
|  |  |  |  |  |  |  |  |
| **FERT, famhx, reaction times** | | |  |  |  |  |  |
|  |  | ***Placebo/Simvastatin*** |  |  |  |  |  |
|  |  | *MD* | *SE* | *p* |  |  |  |
| *Sad* | NoFamHx | -0.02 | 0.12 | 0.87 |  |  |  |
|  | PosFamHx | -0.08 | 0.16 | 0.62 |  |  |  |
| *Fear* | NoFamHx | 0.04 | 0.19 | 0.84 |  |  |  |
|  | PosFamHx | -0.02 | 0.25 | 0.93 |  |  |  |
| *Happy* | NoFamHx | 0.08 | 0.11 | 0.46 |  |  |  |
|  | PosFamHx | -0.03 | 0.14 | 0.85 |  |  |  |
| *Surprise* | NoFamHx | 0.12 | 0.10 | 0.25 |  |  |  |
|  | PosFamHx | -0.11 | 0.13 | 0.43 |  |  |  |
| *Disgust* | NoFamHx | -0.02 | 0.11 | 0.89 |  |  |  |
|  | PosFamHx | -0.20 | 0.14 | 0.15 |  |  |  |
| *Anger* | NoFamHx | -0.01 | 0.13 | 0.93 |  |  |  |
|  | PosFamHx | 0.00 | 0.16 | 0.99 |  |  |  |
| *Neutral* | NoFamHx | 0.02 | 0.12 | 0.87 |  | *F* | *p* |
|  | PosFamHx | -0.11 | 0.16 | 0.50 |  | 0.42 | 0.78 |
|  |  |  |  |  |  |  |  |
| **FERT, famhx, misclassifications** | | |  |  |  |  |  |
|  |  | ***Placebo/Simvastatin*** |  |  |  |  |  |
|  |  | *MD* | *SE* | *p* |  |  |  |
| *Sad* | NoFamHx | -0.50 | 1.82 | 0.79 |  |  |  |
|  | PosFamHx | 6.10 | 2.39 | 0.01 |  |  |  |
| *Fear* | NoFamHx | 0.77 | 1.36 | 0.57 |  |  |  |
|  | PosFamHx | -0.24 | 1.79 | 0.90 |  |  |  |
| *Happy* | NoFamHx | 0.37 | 0.86 | 0.67 |  |  |  |
|  | PosFamHx | -1.47 | 1.13 | 0.20 |  |  |  |
| *Surprise* | NoFamHx | -1.03 | 1.70 | 0.55 |  |  |  |
|  | PosFamHx | -1.03 | 1.70 | 0.55 |  |  |  |
| *Disgust* | NoFamHx | 0.35 | 1.50 | 0.82 |  |  |  |
|  | PosFamHx | -2.82 | 1.97 | 0.16 |  |  |  |
| *Anger* | NoFamHx | 0.40 | 1.33 | 0.76 |  |  |  |
|  | PosFamHx | -2.97 | 1.75 | 0.10 |  |  |  |
| *Neutral* | NoFamHx | -0.36 | 3.62 | 0.92 |  | *F* | *p* |
|  | PosFamHx | -0.86 | 4.75 | 0.86 |  | 1.14 | 0.33 |
|  |  |  |  |  |  |  |  |
| **FERT, famhx, accuracy positive/negative** | | | |  |  |  |  |
|  |  | ***Placebo/Simvastatin*** |  |  |  |  |  |
|  |  | *MD* | *SE* | *p* |  |  |  |
| *Positive* | NoFamHx | 0.56 | 2.30 | 0.81 |  |  |  |
|  | PosFamHx | -5.91 | 2.96 | 0.05 |  |  |  |
| *Negative* | NoFamHx | 0.98 | 2.57 | 0.70 |  | *F* | *p* |
|  | PosFamHx | 0.45 | 2.78 | 0.87 |  | 1.30 | 0.26 |
|  |  | 0.594 | 3.303 | 0.858 |  |  |  |
| **FERT, famhx, d'** | |  |  |  |  |  |  |
|  |  | ***Placebo/Simvastatin*** |  |  |  |  |  |
|  |  | *MD* | *SE* | *p* |  |  |  |
| *Sad* | NoFamHx | 0.00 | 0.01 | 0.76 |  |  |  |
|  | PosFamHx | 0.00 | 0.01 | 0.89 |  |  |  |
| *Fear* | NoFamHx | -0.01 | 0.03 | 0.69 |  |  |  |
|  | PosFamHx | -0.02 | 0.03 | 0.62 |  |  |  |
| *Happy* | NoFamHx | 0.01 | 0.01 | 0.49 |  |  |  |
|  | PosFamHx | -0.02 | 0.01 | 0.06 |  |  |  |
| *Surprise* | NoFamHx | 0.00 | 0.01 | 0.93 |  |  |  |
|  | PosFamHx | -0.02 | 0.01 | 0.15 |  |  |  |
| *Disgust* | NoFamHx | 0.00 | 0.01 | 0.85 |  |  |  |
|  | PosFamHx | 0.03 | 0.02 | 0.11 |  |  |  |
| *Anger* | NoFamHx | 0.01 | 0.01 | 0.55 |  |  |  |
|  | PosFamHx | 0.01 | 0.02 | 0.70 |  |  |  |
| *Neutral* | NoFamHx | 0.00 | 0.02 | 0.99 |  | *F* | *p* |
|  | PosFamHx | -0.03 | 0.02 | 0.21 |  | 0.90 | 0.45 |
|  |  |  |  |  |  |  |  |
| **FERT, famhx, beta** | |  |  |  |  |  |  |
|  |  | ***Placebo/Simvastatin*** |  |  |  |  |  |
|  |  | *MD* | *SE* | *p* |  |  |  |
| *Sad* | NoFamHx | 0.02 | 0.06 | 0.67 |  |  |  |
|  | PosFamHx | -0.22 | 0.07 | 0.01 |  |  |  |
| *Fear* | NoFamHx | -0.08 | 0.07 | 0.27 |  |  |  |
|  | PosFamHx | -0.01 | 0.09 | 0.89 |  |  |  |
| *Happy* | NoFamHx | -0.03 | 0.05 | 0.60 |  |  |  |
|  | PosFamHx | 0.02 | 0.06 | 0.73 |  |  |  |
| *Surprise* | NoFamHx | 0.05 | 0.06 | 0.37 |  |  |  |
|  | PosFamHx | -0.06 | 0.08 | 0.47 |  |  |  |
| *Disgust* | NoFamHx | -0.02 | 0.05 | 0.75 |  |  |  |
|  | PosFamHx | 0.09 | 0.06 | 0.16 |  |  |  |
| *Anger* | NoFamHx | -0.02 | 0.04 | 0.60 |  |  |  |
|  | PosFamHx | 0.08 | 0.06 | 0.16 |  |  |  |
| *Neutral* | NoFamHx | 0.05 | 0.16 | 0.75 |  | *F* | *p* |
|  | PosFamHx | 0.11 | 0.20 | 0.58 |  | 0.98 | 0.38 |

***ECAT***

| **ECAT, correct** |  |  |  |  |  |  |  |
| --- | --- | --- | --- | --- | --- | --- | --- |
|  | **Placebo** |  | **Simvastatin** |  |  |  |  |
|  | N= 25 |  | N= 27 |  |  |  |  |
| Positive | 94.00 | 1.39 | 94.82 | 1.34 |  | *F* | *p* |
| Negative | 97.20 | 1.00 | 94.63 | 0.96 |  | 2.90 | 0.10 |
|  |  |  |  |  |  |  |  |
| **ECAT, reaction times** |  |  |  |  |  |  |  |
|  | **Placebo** |  | **Simvastatin** |  |  |  |  |
|  | N= 26 |  | N= 27 |  |  |  |  |
| Positive | 0.88 | 0.05 | 0.99 | 0.04 |  | *F* | *p* |
| Negative | 1.00 | 0.05 | 1.07 | 0.04 |  | 2.48 | 0.12 |

***EREC***

| **EREC, correct** |  |  |  |  |  |  |  |
| --- | --- | --- | --- | --- | --- | --- | --- |
|  | **Placebo** |  | **Simvastatin** |  |  |  |  |
|  | N= 26 |  | N= 27 |  |  |  |  |
| Positive | 5.46 | 0.39 | 6.11 | 0.38 |  | *F* | *p* |
| Negative | 4.12 | 0.45 | 4.89 | 0.44 |  | 0.04 | 0.84 |
|  |  |  |  |  |  |  |  |
| **EREC, incorrect** |  |  |  |  |  |  |  |
|  | **Placebo** |  | **Simvastatin** |  |  |  |  |
|  | N= 26 |  | N= 27 |  |  |  |  |
| Positive | 1.77 | 0.30 | 2.70 | 0.29 |  | *F* | *p* |
| Negative | 1.19 | 0.24 | 1.15 | 0.24 |  | 4.86 | 0.03 |

|  | *MD* | *SE* | *F* | *p* | *η^2^* |
| --- | --- | --- | --- | --- | --- |
| *Positive* | -0.93 | 0.42 | 4.99 | 0.03 | 0.09 |

***EMEM***

| **EMEM, correct** |  |  |  |  |  |  |  |
| --- | --- | --- | --- | --- | --- | --- | --- |
|  | **Placebo** |  | **Simvastatin** |  |  |  |  |
|  | N= 24 |  | N= 27 |  |  |  |  |
| Positive | 79.98 | 1.65 | 81.93 | 1.55 |  | *F* | *p* |
| Negative | 81.81 | 1.55 | 83.65 | 1.47 |  | 0.00 | 0.96 |
|  |  |  |  |  |  |  |  |
| **EMEM, reaction times** |  |  |  |  |  |  |  |
|  | **Placebo** |  | **Simvastatin** |  |  |  |  |
|  | N= 24 |  | N= 27 |  |  |  |  |
| Positive | 1.07 | 0.05 | 1.13 | 0.04 |  | *F* | *p* |
| Negative | 1.12 | 0.05 | 1.17 | 0.04 |  | 0.04 | 0.85 |
|  |  |  |  |  |  |  |  |
| **EMEM, misclassification** | |  |  |  |  |  |  |
|  | **Placebo** |  | **Simvastatin** |  |  |  |  |
|  | N= 24 |  | N= 27 |  |  |  |  |
| Positive | 18.80 | 1.70 | 17.34 | 1.60 |  | *F* | *p* |
| Negative | 17.48 | 1.53 | 15.90 | 1.44 |  | 0.00 | 0.96 |

***FDOT***

| **FDOT, masked, congruent, accuracy** |  |  |  |  |  |  |  |
| --- | --- | --- | --- | --- | --- | --- | --- |
|  | **Placebo** |  | **Simvastatin** |  |  |  |  |
|  | N= 26 |  | N= 27 |  |  |  |  |
| Happy | 98.08 | 1.09 | 97.22 | 1.07 |  | *F* | *p* |
| Fear | 94.44 | 1.70 | 95.47 | 1.67 |  | 0.51 | 0.48 |
|  |  |  |  |  |  |  |  |
| **FDOT, unmasked, congruent, accuracy** |  |  |  |  |  |  |  |
|  | **Placebo** |  | **Simvastatin** |  |  |  |  |
|  | N= 26 |  | N= 27 |  |  |  |  |
| Happy | 96.64 | 1.07 | 97.22 | 1.05 |  | *F* | *p* |
| Fear | 95.73 | 1.32 | 95.47 | 1.29 |  | 0.17 | 0.68 |
|  |  |  |  |  |  |  |  |
| **FDOT, masked, congruent, reaction times** |  |  |  |  |  |  |  |
|  | **Placebo** |  | **Simvastatin** |  |  |  |  |
|  | N= 26 |  | N= 27 |  |  |  |  |
| Happy | 0.60 | 0.02 | 0.60 | 0.02 |  | *F* | *p* |
| Fear | 0.58 | 0.02 | 0.57 | 0.02 |  | 0.75 | 0.39 |
|  |  |  |  |  |  |  |  |
| **FDOT, unmasked, congruent, reaction times** |  |  |  |  |  |  |  |
|  | **Placebo** |  | **Simvastatin** |  |  |  |  |
|  | N= 26 |  | N= 27 |  |  |  |  |
| Happy | 0.62 | 0.02 | 0.59 | 0.02 |  | *F* | *p* |
| Fear | 0.61 | 0.02 | 0.59 | 0.02 |  | 0.04 | 0.84 |
|  |  |  |  |  |  |  |  |
| **FDOT, masked, vigilance** |  |  |  |  |  |  |  |
|  | **Placebo** |  | **Simvastatin** |  |  |  |  |
|  | N= 26 |  | N= 27 |  |  |  |  |
| Happy | -0.03 | 0.01 | -0.05 | 0.01 |  | *F* | *p* |
| Fear | 0.03 | 0.01 | 0.02 | 0.01 |  | 0.20 | 0.66 |
|  |  |  |  |  |  |  |  |
| **FDOT, unmasked, vigilance** |  |  |  |  |  |  |  |
|  | **Placebo** |  | **Simvastatin** |  |  |  |  |
|  | N= 26 |  | N= 27 |  |  |  |  |
| Happy | -0.02 | 0.01 | -0.02 | 0.01 |  | *F* | *p* |
| Fear | 0.01 | 0.01 | 0.04 | 0.01 |  | 0.97 | 0.33 |

***PILT***

| **PILT, total won** |  |  |  |  |  |  |  |
| --- | --- | --- | --- | --- | --- | --- | --- |
|  | **Placebo** |  | **Simvastatin** |  |  |  |  |
|  | N= 26 |  | N= 27 |  |  | *F* | *p* |
|  | 5.20 | 0.31 | 5.11 | 0.30 |  | 0.04 | 0.84 |
|  |  |  |  |  |  |  |  |
| **PILT, rew** |  |  |  |  |  |  |  |
|  | **Placebo** |  | **Simvastatin** |  |  |  |  |
|  | N= 26 |  | N= 27 |  |  | *F* | *p* |
|  | 0.82 | 0.04 | 0.81 | 0.04 |  | 0.13 | 0.72 |
|  |  |  |  |  |  |  |  |
| **PILT, rew t16-30** |  |  |  |  |  |  |  |
|  | **Placebo** |  | **Simvastatin** |  |  |  |  |
|  | N= 26 |  | N= 27 |  |  | *F* | *p* |
|  | 0.85 | 0.04 | 0.85 | 0.04 |  | 0.00 | 0.95 |
|  |  |  |  |  |  |  |  |
| **PILT, won** |  |  |  |  |  |  |  |
|  | **Placebo** |  | **Simvastatin** |  |  |  |  |
|  | N= 26 |  | N= 27 |  |  | *F* | *p* |
|  | 2.43 | 0.06 | 2.39 | 0.06 |  | 0.18 | 0.67 |
|  |  |  |  |  |  |  |  |
| **PILT, won t16-30** |  |  |  |  |  |  |  |
|  | **Placebo** |  | **Simvastatin** |  |  |  |  |
|  | N= 26 |  | N= 27 |  |  | *F* | *p* |
|  | 1.22 | 0.03 | 1.22 | 0.03 |  | 0.00 | 0.99 |
|  |  |  |  |  |  |  |  |
| **PILT, los** |  |  |  |  |  |  |  |
|  | **Placebo** |  | **Simvastatin** |  |  |  |  |
|  | N= 26 |  | N= 26 |  |  | *F* | *p* |
|  | 0.25 | 0.03 | 0.23 | 0.03 |  | 0.19 | 0.67 |
|  |  |  |  |  |  |  |  |
| **PILT, los t16-30** |  |  |  |  |  |  |  |
|  | **Placebo** |  | **Simvastatin** |  |  |  |  |
|  | N= 26 |  | N= 27 |  |  | *F* | *p* |
|  | 0.22 | 0.04 | 0.17 | 0.04 |  | 0.82 | 0.37 |
|  |  |  |  |  |  |  |  |
| **PILT, lost** |  |  |  |  |  |  |  |
|  | **Placebo** |  | **Simvastatin** |  |  |  |  |
|  | N= 26 |  | N= 27 |  |  | *F* | *p* |
|  | -1.69 | 0.06 | -1.68 | 0.06 |  | 0.01 | 0.92 |
|  |  |  |  |  |  |  |  |
| **PILT, lost t16-30** |  |  |  |  |  |  |  |
|  | **Placebo** |  | **Simvastatin** |  |  |  |  |
|  | N= 26 |  | N= 27 |  |  | *F* | *p* |
|  | -0.84 | 0.03 | -0.80 | 0.03 |  | 0.56 | 0.46 |
|  |  |  |  |  |  |  |  |
|  |  |  |  |  |  |  |  |
| **PILT, rew switch** |  |  |  |  |  |  |  |
|  | **Placebo** |  | **Simvastatin** |  |  |  |  |
|  | N= 22 |  | N= 25 |  |  | *F* | *p* |
|  | 0.09 | 0.02 | 0.10 | 0.02 |  | 0.24 | 0.63 |
|  |  |  |  |  |  |  |  |
| **PILT, rew switch t16-30** |  |  |  |  |  |  |  |
|  | **Placebo** |  | **Simvastatin** |  |  |  |  |
|  | N= 22 |  | N= 25 |  |  | *F* | *p* |
|  | 0.04 | 0.02 | 0.07 | 0.02 |  | 1.04 | 0.31 |
|  |  |  |  |  |  |  |  |
| **PILT, los switch** |  |  |  |  |  |  |  |
|  | **Placebo** |  | **Simvastatin** |  |  |  |  |
|  | N= 26 |  | N= 27 |  |  | *F* | *p* |
|  | 0.09 | 0.02 | 0.10 | 0.02 |  | 0.01 | 0.91 |
|  |  |  |  |  |  |  |  |
| **PILT, los switch t16-30** |  |  |  |  |  |  |  |
|  | **Placebo** |  | **Simvastatin** |  |  |  |  |
|  | N= 26 |  | N= 27 |  |  | *F* | *p* |
|  | 0.25 | 0.04 | 0.24 | 0.04 |  | 0.03 | 0.88 |
|  |  |  |  |  |  |  |  |
| **PILT, rew switch/rew switch t16-30** |  |  |  |  |  |  |  |
|  | **Placebo** |  | **Simvastatin** |  |  |  |  |
|  | N= 22 |  | N= 25 |  |  | *F* | *p* |
| Rew switch | 0.09 | 0.02 | 0.10 | 0.02 |  | 0.97 | 0.33 |
| Rew switch t16-30 | 0.04 | 0.02 | 0.07 | 0.02 |  | 0.97 | 0.33 |
|  |  |  |  |  |  |  |  |
| **PILT, los switch/los switch t16-30** |  |  |  |  |  |  |  |
|  | **Placebo** |  | **Simvastatin** |  |  |  |  |
|  | N= 26 |  | N= 27 |  |  | *F* | *p* |
| Los switch | 0.28 | 0.03 | 0.27 | 0.03 |  | 0.04 | 0.85 |
| Los switch t16-30 | 0.25 | 0.04 | 0.24 | 0.04 |  | 0.04 | 0.85 |

***AVLT***

| **AVLT, total correct** |  |  |  |  |  |  |  |
| --- | --- | --- | --- | --- | --- | --- | --- |
|  | **Placebo** |  | **Simvastatin** |  |  |  |  |
|  | N= 26 |  | N= 27 |  |  | *F* | *p* |
|  | 58.19 | 1.42 | 60.26 | 1.40 |  | 1.07 | 0.31 |
|  |  |  |  |  |  |  |  |
| **AVLT, short delay correct** |  |  |  |  |  |  |  |
|  | **Placebo** |  | **Simvastatin** |  |  |  |  |
|  | N= 26 |  | N= 27 |  |  | *F* | *p* |
|  | 12.39 | 0.38 | 13.11 | 0.37 |  | 1.85 | 0.18 |
|  |  |  |  |  |  |  |  |
| **AVLT, long delay correct** |  |  |  |  |  |  |  |
|  | **Placebo** |  | **Simvastatin** |  |  |  |  |
|  | N= 26 |  | N= 27 |  |  | *F* | *p* |
|  | 12.65 | 0.38 | 13.11 | 0.38 |  | 0.73 | 0.40 |
|  |  |  |  |  |  |  |  |
| **AVLT, intrusions** |  |  |  |  |  |  |  |
|  | **Placebo** |  | **Simvastatin** |  |  |  |  |
|  | N= 26 |  | N= 27 |  |  | *F* | *p* |
|  | 1.28 | 0.39 | 1.31 | 0.39 |  | 0.00 | 0.96 |
|  |  |  |  |  |  |  |  |
| **AVLT, repetitions** |  |  |  |  |  |  |  |
|  | **Placebo** |  | **Simvastatin** |  |  |  |  |
|  | N= 26 |  | N= 26 |  |  | *F* | *p* |
|  | 5.19 | 0.78 | 3.39 | 0.78 |  | 2.70 | 0.11 |
|  |  |  |  |  |  |  |  |
| **AVLT, recognition hits** |  |  |  |  |  |  |  |
|  | **Placebo** |  | **Simvastatin** |  |  |  |  |
|  | N= 25 |  | N= 26 |  |  | *F* | *p* |
|  | 14.36 | 0.17 | 14.50 | 0.16 |  | 0.36 | 0.55 |
|  |  |  |  |  |  |  |  |
| **AVLT, recognition false alarm** |  |  |  |  |  |  |  |
|  | **Placebo** |  | **Simvastatin** |  |  |  |  |
|  | N= 25 |  | N= 27 |  |  | *F* | *p* |
|  | 0.40 | 0.12 | 0.22 | 0.11 |  | 1.23 | 0.27 |

##### S5 – Outcome measures of the Facial Expression Recognition Task

***FERT.*** Effects of simvastatin on facial expression recognition (FERT). Results for accuracy, reaction times, and misclassifications for all seven facial expressions are shown. Values are means ± standard error of the mean bars, an asterisk (*) represents a statistically significant difference between the simvastatin (grey) and placebo (white) groups.


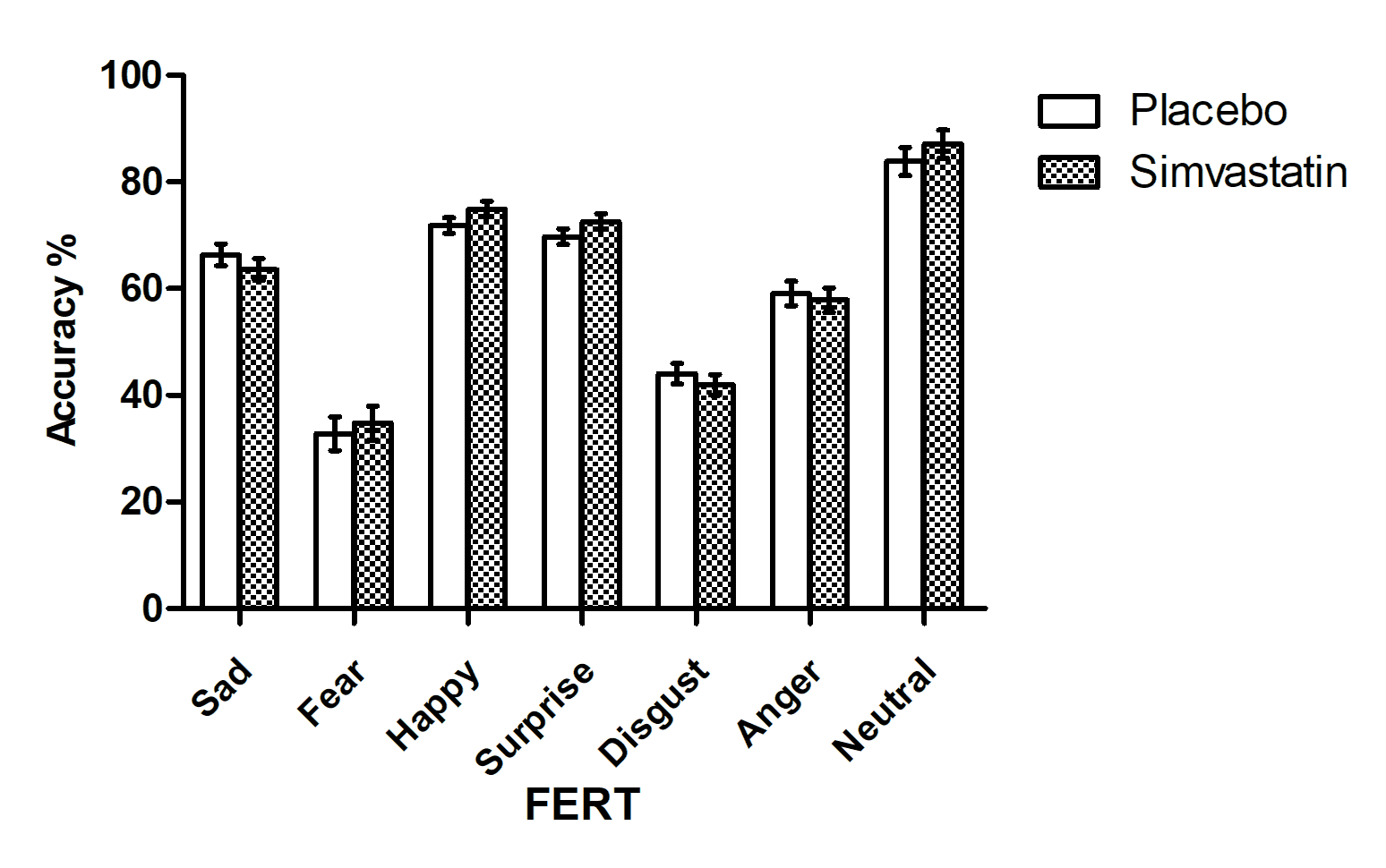

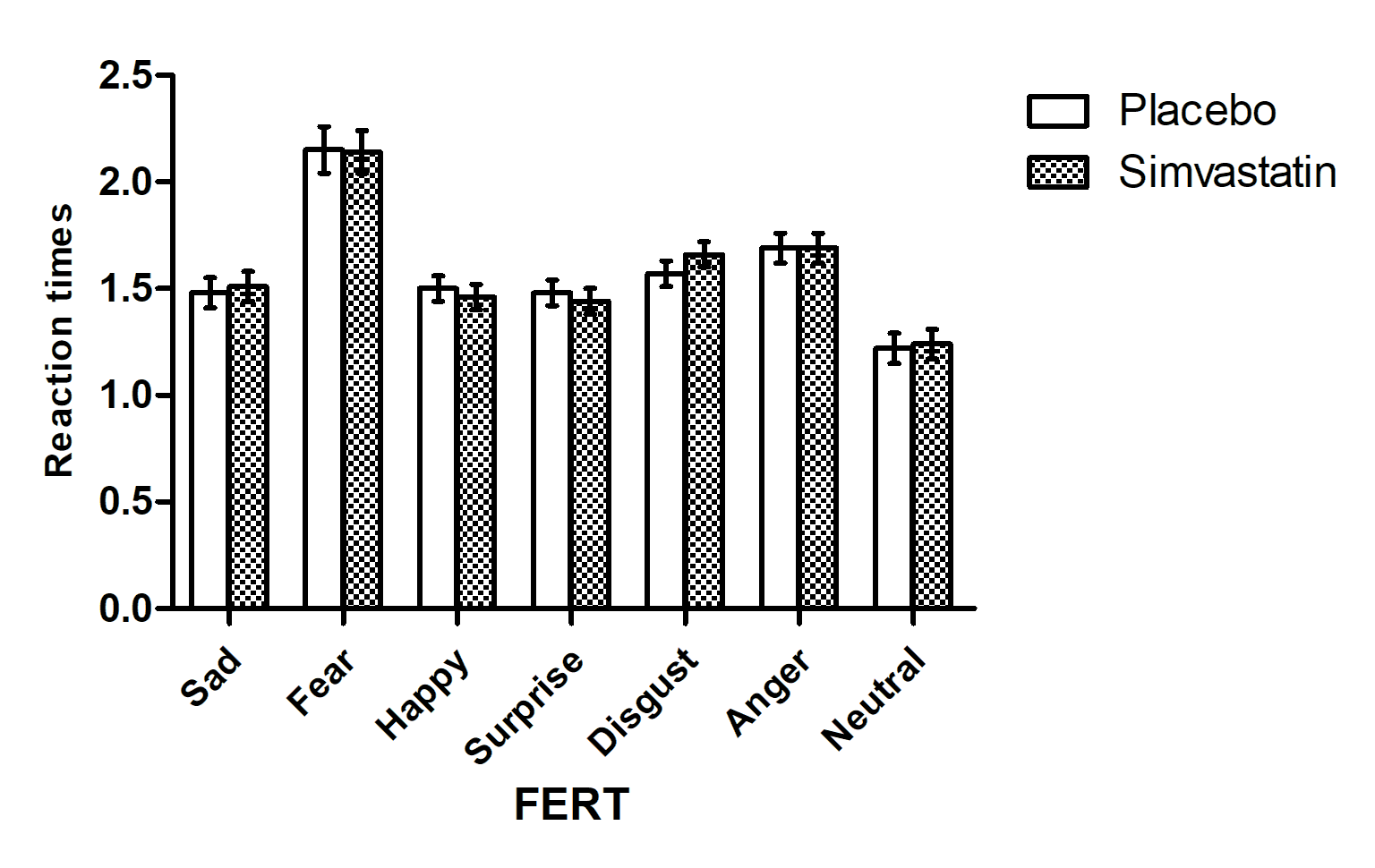

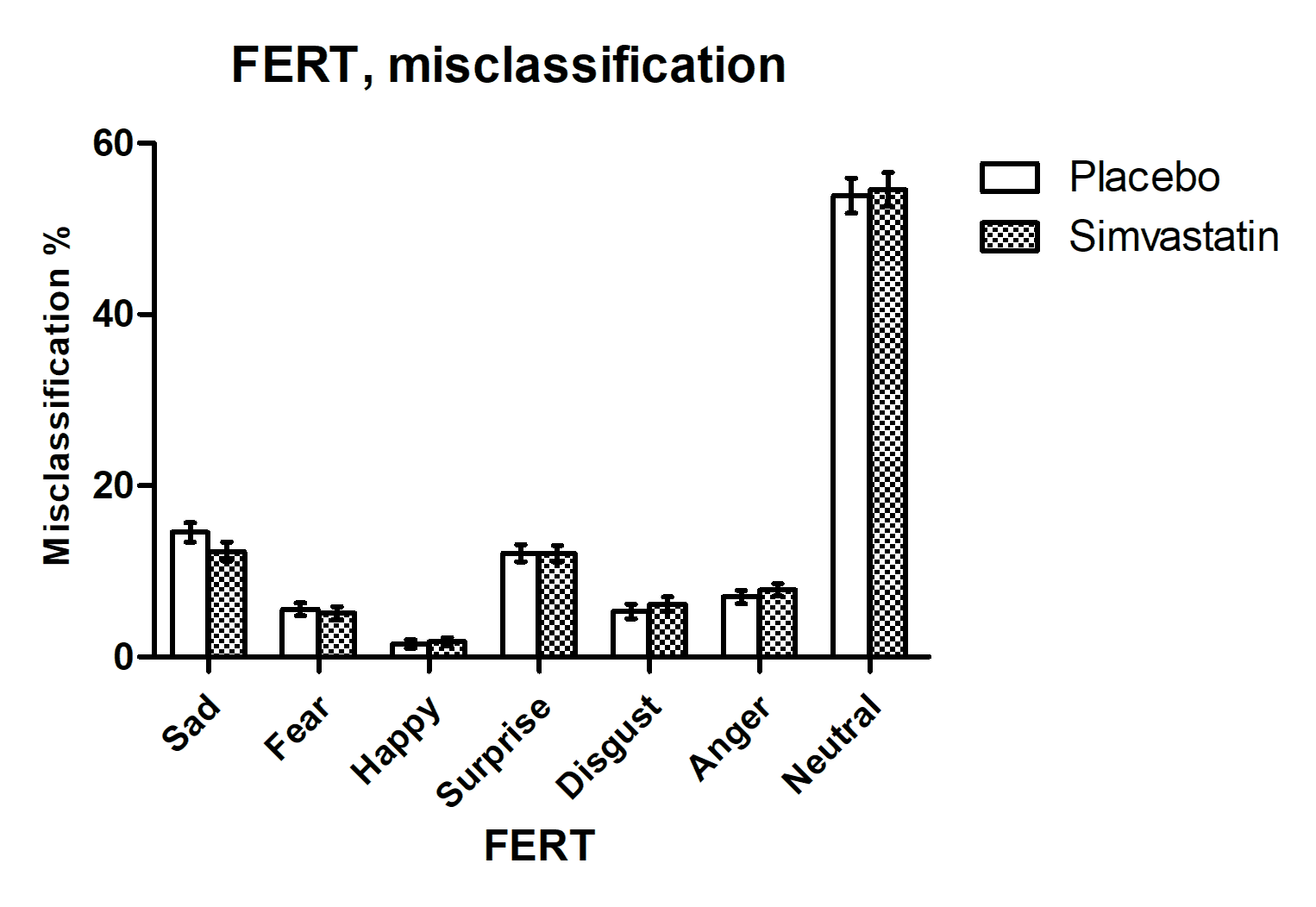


***FERT, females’ subgroup.*** Effects of simvastatin on facial expression recognition in the females’ subgroup. Results for misclassifications for all seven facial expressions, and for positive faces misclassified as sad facial expressions are shown. Values are means ± standard error of the mean bars, an asterisk (*) represents a statistically significant difference between the simvastatin (grey) and placebo (white) groups.

*
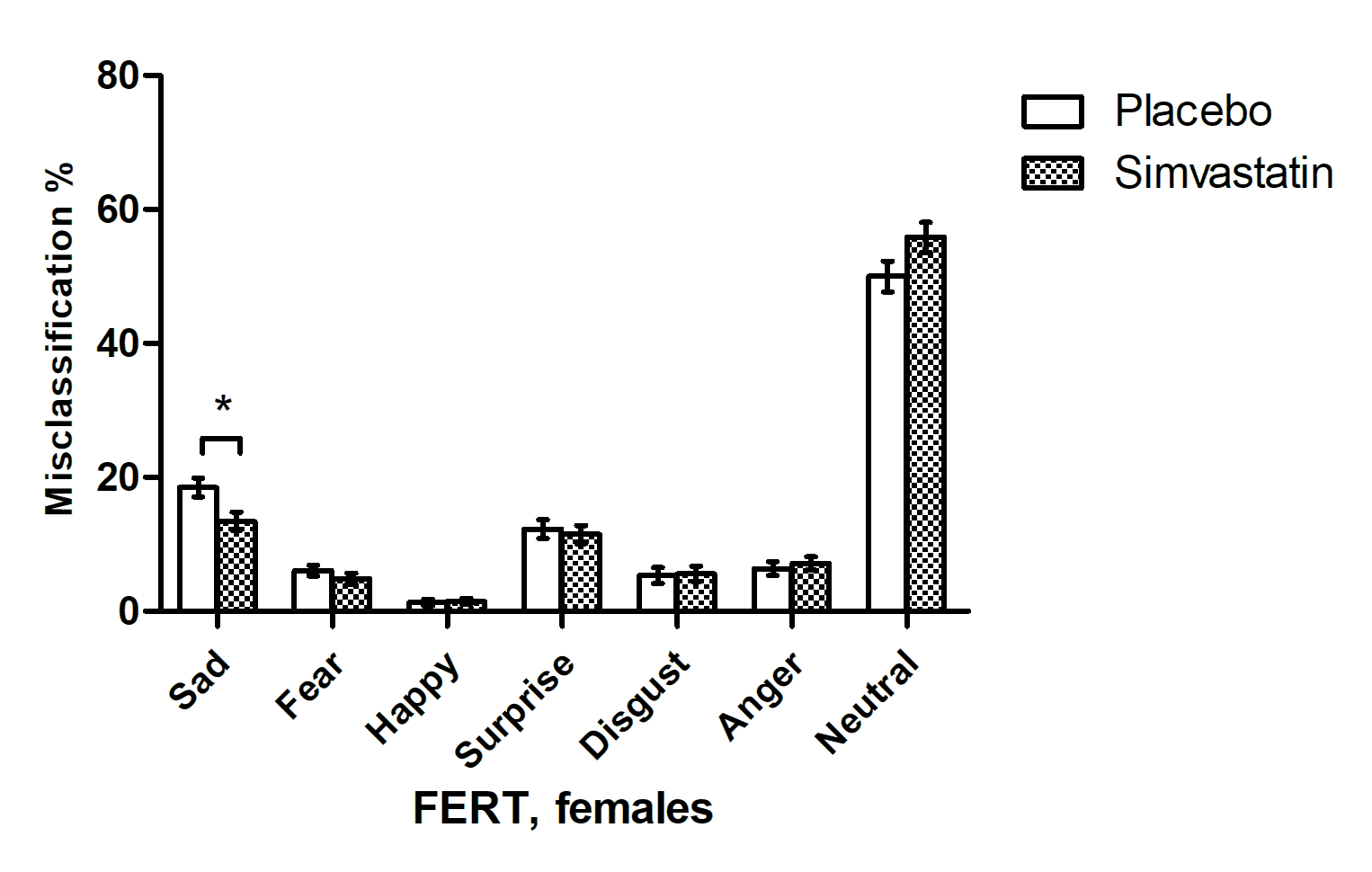
*

*
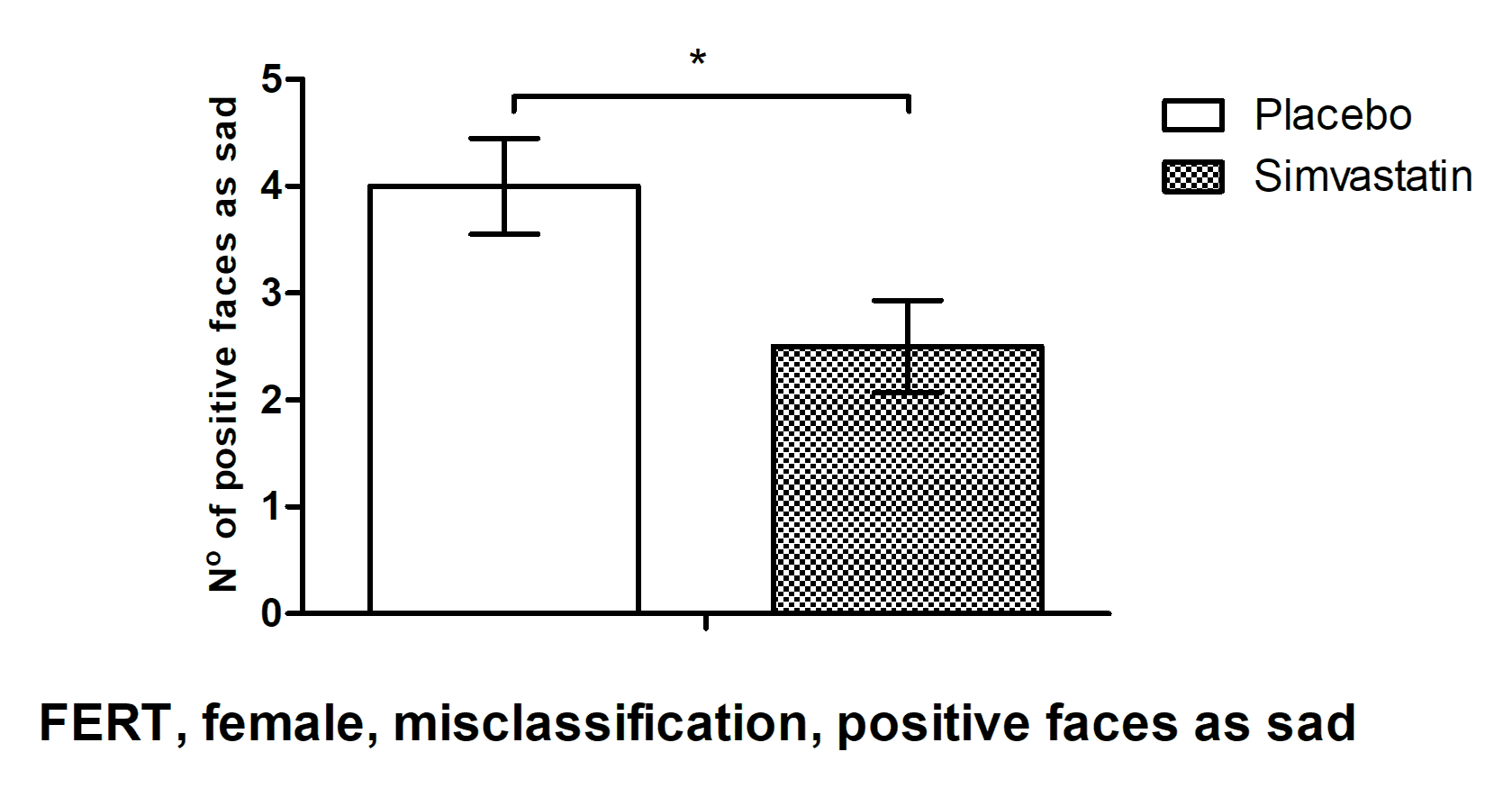
*

##### S6 – Probabilistic instrumental learning task, learning curves

*
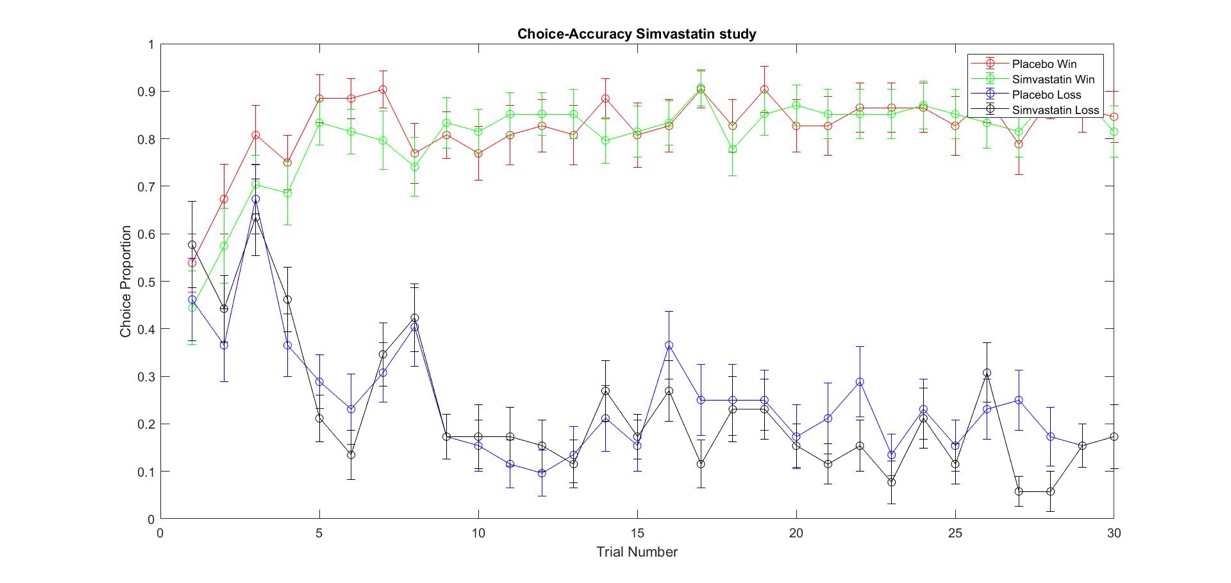
*
